# Ferroptosis-Related Genes in IgA Nephropathy: Screening for Potential Targets of the Mechanism

**DOI:** 10.1155/2024/8851124

**Published:** 2024-08-14

**Authors:** Wenhui Zhu, Yao Chen, Jing Xiao, Chuchu Cheng, Guijie Ma, Yang Wang, Yonggang Zhang, Ming Chen

**Affiliations:** ^1^ Department of Renal Division Heilongjiang Academy of Chinese Medicine Sciences, Harbin, China; ^2^ College of Traditional Chinese Medicine Changchun University of Chinese Medicine, Changchun, China; ^3^ Department of Renal Division First People's Hospital of Qiqihar City, Qiqihar, China

**Keywords:** ATF3, bioinformatics, ferroptosis, IgAN, IL-17, JUN

## Abstract

**Aims:** Exploring key genes and potential molecular pathways of ferroptosis in immunoglobulin A nephropathy (IgAN).

**Methods:** The IgAN datasets and ferroptosis-related genes (FRGs) were obtained in the Gene Expression Omnibus (GEO) and FerrDb database. Differentially expressed genes (DEGs) were identified using R software and intersected with FRGs to obtain differentially expressed FRGs (DE-FRGs). After that, the Kyoto Encyclopedia of Genes and Genomes (KEGG) pathway enrichment analysis (PEA) and Gene Ontology (GO) functional annotation were performed on DE-FRGs. In the Search Tool for the Retrieval of Interacting Genes (STRING) website, we construct a protein–protein interaction (PPI) network. The PPI network was further investigated with screening hub genes with Cytoscape software. The core genes were then subjected to gene set enrichment analysis (GSEA). Finally, the samples were analyzed for immune infiltration in R, and the correlation between hub genes and immune cells was analyzed.

**Results:** A total of 347 DEGs were identified. CD44, CDO1, CYBB, IL1B, RRM2, AKR1C1, activated transcription factor-3 (ATF3), CDKN1A, GDF15, JUN, MGST1, MIOX, MT1G, NR4A1, PDK4, TNFAIP3, and ZFP36 were determined as DE-FRGs. JUN, IL1B, and ATF3 were then screened as hub genes. GSEA and immune infiltration analysis revealed that the hub genes were closely associated with immune inflammatory responses such as NOD-like receptor signaling, IL-17 signaling, and TNF signaling.

**Conclusions:** Our results show that JUN and ATF3 are possibly critical genes in the process of IgAN ferroptosis and may be related with immune cell infiltration.

## 1. Introduction

Immunoglobulin A nephropathy (IgAN) is an autoimmune disease [1]. IgAN is also known as Berger's disease [2, 3], which is characterized pathologically by IgA immune complex deposition in the glomerular mesangial region [4]. The clinical presentation of IgAN varies from asymptomatic microscopic hematuria to nephrotic syndrome, depending on factors such as age and type of pathology, which undoubtedly increases the burden of diagnosing and treating the disease. In Asia, IgAN is the most frequent glomerulonephritis and accounts for 40% of all types of glomerulonephritis [5], with an incidence of more than 2.5 per 100,000 [6]. It is notable that patients with IgAN have an increased risk of death due to a lack of early diagnosis and special effective treatment [7]. Although the pathogenesis has been characterized as a multi-“hit” process including galactose-deficient IgA1, antiglycan response, formation and deposition of IgA1-containing immune complexes, and mechanisms of immune complex–mediated tissue injury [8], the major pathways, specific key molecules, and new genetic susceptibility loci remain to be further explored [9]. Therefore, there is an urgent need to propose a new idea to study the potential or key candidate genes of IgAN in order to understand the molecular mechanism of IgAN.

Previously, researchers identified an iron-dependent type of programmed cell death named ferroptosis [10], which is mainly manifested by excess iron-mediated accretion of reactive oxygen species (ROS) in intracellular lipids and imbalance of redox reactions [11]. Ferritin dysregulation has been linked to a number of biological processes (BPs) in mammals, including degenerative diseases (such as Huntington's, Parkinson's, and Alzheimer's), traumatic brain injury, carcinogenesis, intracerebral hemorrhage, ischemia-reperfusion injury, stroke, and kidney degeneration [12]. In addition, IgAN hematuria is rich in erythrocytes, and renal tubular epithelial cells have the function of phagocytosis and degradation of erythrocytes, which leads to an increase in the production of Fe2+ from the metabolism of hemoglobin, which reacts with H_2_O_2_ to produce ROS that damage renal cells [13]. There is growing evidence that ferroptosis plays a crucial role in IgAN [14, 15]. Therefore, an in-depth exploration of ferroptosis is crucial for identifying precise mechanisms underlying IgAN, accurately controlling the disease's progression, and developing novel approaches for its clinical prevention and treatment.

In this study, we mined public databases using bioinformatic methods to identify ferroptosis-related genes (FRGs) and pathways that are significant to IgAN. These genes and pathways can serve as potential therapeutic targets and biomarkers and provide clues for future research and treatment of IgAN.

## 2. Materials and Methods

### 2.1. Data Collection

In this study, we used three RNA expression microarray datasets (GSE93798, GSE115857, and GSE35487) obtained from the Gene Expression Omnibus (GEO) database (http://www.ncbi.nlm.nih.gov/geo/). Their gene annotation platforms are GPL22945 (Affymetrix Human Genome U133 Plus 2.0 Array), GPL14951 (Illumina HumanHT-12 WG-DASL V4.0 R2 expression beadchip), and GPL96 (Affymetrix Human Genome U133A Array). The GSE93798 contained 22 normal control samples and 20 IgAN samples; GSE115857 contained 55 IgAN samples and 7 normal control samples; and GSE35487 contained 25 IgAN samples and 7 normal control samples. The GSE93798 dataset was used to determine the key candidate genes. Another two independent datasets, GSE115857 and GSE35487, were used for the validation of key candidate genes.

A total of 396 FRGs, including drivers, suppressors, and markers of ferroptosis, were downloaded from the public FerrDb database (http://www.zhounan.org/ferrdb). The genes were filtered for “human,” and duplicates were removed. The detailed genes are shown in Table S1. Furthermore, the research was carried out in compliance with the Helsinki Declaration (as revised in 2013).

### 2.2. Analysis of Variances

The R software package “limma” was used to analyze the microarray data in order to identify differentially expressed genes (DEGs) between the control and IgAN sample, based on classical Bayesian algorithm. First, probes were annotated using an annotation platform file. Probe sets without known annotation, mapped to multiple gene IDs, or did not map to any gene ID were removed. In cases where a gene corresponds to a plurality of probe sets, the maximum value was used as the gene expression value. DEGs were selected according to the following criterions: |log_2_FC| > 1 and adjusted probability value (*p* value) < 0.05, where FC is the fold change. Based on these results, heat maps and volcano plots were drawn with the “ggplot2” package in R.

### 2.3. Acquisition of Ferroptosis-Related DEGs

Intersecting genes from FRGs and DEGs were designated as differentially expressed FRGs (DE-FRGs). Thereafter, the DE-FRGs were displayed in a Venn diagram using the “Venndiagram” package in R. DE-FRGs were used in later analyses.

### 2.4. Functional Enrichment Analysis of DE-FRGs

R software was used to perform a Gene Ontology (GO) enrichment analysis of DE-FRGs, which included an analysis of the categories of BP, cellular component (CC), and molecular function (MF). The Kyoto Encyclopedia of Genes and Genomes (KEGG) pathway analysis of DE-FRGs was conducted by R software. They both use the “cluster profiler” package in R. KEGG was performed mainly for pathway analysis. We were able to acquire a better grasp of the probable roles of DE-FRGs using GO and KEGG analysis. Significant pathways and GO results with *p* value < 0.05 and gene count > 2 were chosen. Bubble plot and histograms of top 10 KEGG and GO enrichments were plotted with “ggplot2” package in R.

### 2.5. Construction of Protein–Protein Interaction (PPI) Network and Screening of Hub Genes

The Search Tool for the Retrieval of Interacting Genes (STRING) (https://string-db.org/) database was utilized to analyze the interactions of the distinct DE-FRGs. The DE-FRG PPI network was created by limiting the study species to “human” (*Homo sapiens*) and setting the minimal interaction score to medium confidence (0.400), concealing unconnected points, and leaving the remaining parameters at their default values. Then, the STRING interaction file was exported and saved in a TSV format. Data were entered into Cytoscape 3.9.1 software (National Institute of General Medical Sciences, Maryland, United States) for processing and visualization, as well as to investigate which genes could serve as hub genes. The hub genes were selected by betweenness centrality (BC), which was a measurement of diagram centrality based on the shortest path by utilizing the cytoNCA plug-in. BC is a measure of the centrality and importance of a node in a network; nodes with high median centrality can better control the flow of information and connect different parts of the network; it is an effective tool to measure the node's “control power.”

### 2.6. Gene Set Enrichment Analysis (GSEA)

This analysis is performed using the “clusterprofiler” package in R. GSEA is a statistical method for assessing the significance of a particular set of genes [16]. We explored the correlation between the hub genes and other individual genes in the GSE93798 dataset. High- and low-expression categories resulted from this analysis, allowing us to investigate the pathways associated with the hub genes in more detail. This estimate showed the important distinctions between the high and low hub gene groupings that we had previously seen. The phenotypic labels used were the expression levels of the hub genes for genomic alignments, where 1000 replicates were conducted for each study. To determine its enrichment in the gene set, the KEGG signaling pathway set was implemented as a predetermined set. Each hub gene's specific enrichment results were merged into Table S2. The R “enrichplot” package was used to generate visual gene set enrichment maps with annotations. Genomes with an FDR < 0.05 are deemed substantially enriched.

### 2.7. Immune Infiltration Analysis

To examine the immune infiltration landscape of IgAN, single-gene GSEA (ssGSEA) was done using the R package “GSVA” to assess the amount of immune infiltrates in a sample based on the expression levels of immune cell–specific marker genes. The GSVA function is a pathway enrichment analysis (PEA) tool based on a nonparametric approach. We obtained marker genes for the immune cell types from a published paper by Charoentong et al. [17]. Additionally, we have further explored the relationship between immune cells and hub genes. The Spearman rank correlation coefficient was calculated with R software using the “corrplot” package to assess the relationship between immune cells and hub genes. We used the R “ggplot2” package to show the findings.

### 2.8. Statistical Analysis

Student's *t*-test was performed to compare the two groups. The association between 17 DE-FRGs was determined using the Pearson correlation analysis. The *p* < 0.05 was considered as statistically significant. All analysis and visualization were performed in R.

## 3. Results

### 3.1. Acquisition of DEGs

**Figure 1:**
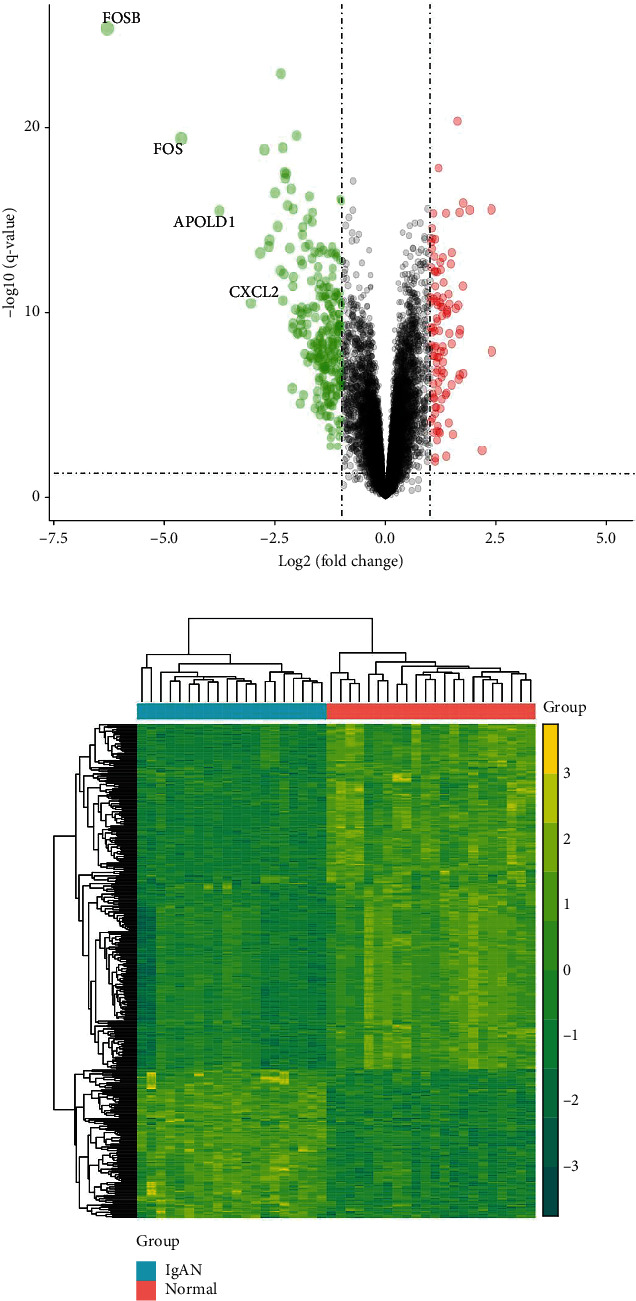
Differentially expressed gene (DEG) analysis on IgA nephropathy. (a) Volcanic plots of DEGs. Red represents upregulated DEGs, green represents downregulated DEGs, and black represents genes, which are not differentially expressed. (b) Heat map of the DEGs.

Gene expression profiles of GSE93798 were used to identify DEGs between the control and IgAN samples. The gene dataset included 20 IgA nephropathy patients and 22 normal population samples. Differential expression analysis was done using the R “limma” package, with filtering criteria of *p* < 0.05 and |log_2_FC| > 1. Our findings revealed several interesting differences in gene expression levels between the control and IgAN groups. The volcano plot showed that 347 genes were differently expressed in the IgAN patients as compared to the normal population, including 104 upregulated genes and 243 downregulated genes (Figure 1(a)). Furthermore, Figure 1(b) depicts heat maps for the dataset, indicating improved sample grouping and increased confidence. Normalization and cross-compatibility of data were also evaluated. The selected samples were centralized and numerically distributed to standard, as shown in Figure S1, confirming the cross comparability and good quality of the microarray data.

### 3.2. Identification of DE-FRGs

**Figure 2:**
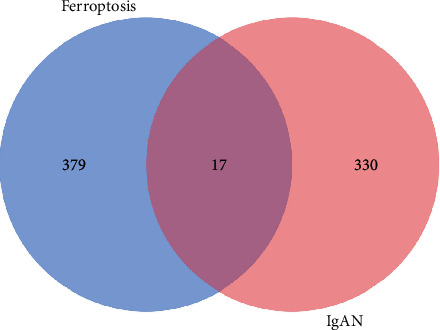
Venn diagram of ferroptosis-related DEGs.

A total of 396 FRGs were retrieved from FerrDb database to explore FRGs differently expressed in IgA nephropathy. Duplicate genes were removed, and “human” was used as the screening condition. The intersection of the FRGs and DEGs produced 17 overlapping genes, including CD44, CDO1, CYBB, IL1B, RRM2, AKR1C1, activated transcription factor-3 (ATF3), CDKN1A, GDF15, JUN, MGST1, MIOX, MT1G, NR4A1, PDK4, TNFAIP3, and ZFP36, which were eventually included in future studies. Finally, these genes were visualized with Venn diagrams (Figure 2).

### 3.3. Functional Enrichment Analysis of DE-FRGs

**Figure 3:**
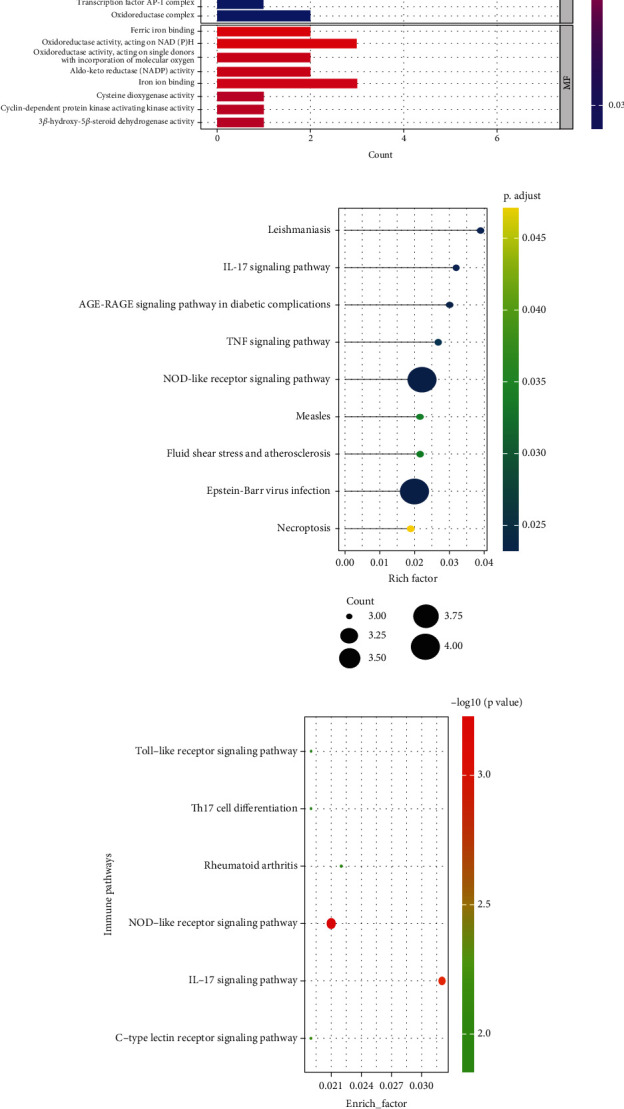
Functional analyses for the DE-FRGs. (a) The result of GO analysis. (b) The significantly enriched KEGG pathways. (c) Enrichment analysis of immune characteristic gene sets.

We investigated the potential biological roles of DE-FRGs by GO enrichment and KEGG pathway analysis. The top 10 clusters of each functional enrichment analysis result are displayed in Figure 3. Consequently, GO enrichment analyses of DE-FRGs indicated that the top three enriched BPs were response to nutrient levels, response to lipopolysaccharide, and response to molecules of bacterial origin (Figure 3(a)); the top three enriched CCs were the ribonucleoside-diphosphate reductase complex, macrophage migration inhibitory factor receptor complex, and PCNA-p21 complex (Figure 3(a)); and the top three enriched MFs were ferric iron binding; oxidoreductase activity, acting on NAD(P)H; and oxidoreductase activity, acting on single donors with incorporation of molecular oxygen (Figure 3(a)). In addition, the KEGG enrichment analysis revealed that these genes were mostly associated with the NOD-like receptor signaling pathway, leishmaniasis, Epstein-Barr virus infection, IL-17 signaling pathway, AGE-RAGE signaling pathway in diabetic complications, TNF signaling pathway, measles, fluid shear stress and atherosclerosis, and necroptosis (Figure 3(b)). Notably, two of the above signaling pathways are immunoinflammatory. Further investigation revealed that DE-FRGs were significantly enriched in various immune inflammation–related pathways (Figure 3(c)). These findings suggest that DE-FRGs may play a role in IgAN through interactions between cytokines, immune cells, and necrosis.

### 3.4. Construction of PPI Network and Obtainment of Hub Genes

**Figure 4:**
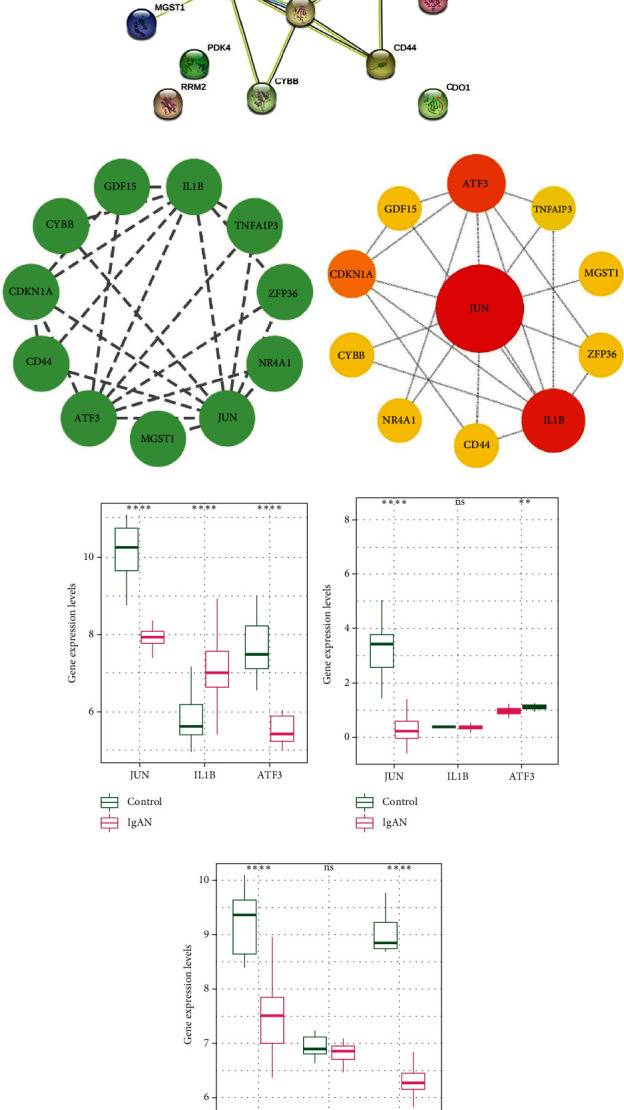
(a) The protein–protein interaction network. (b) PPI network diagram with unconnected targets hidden. (c) Gene set network diagram of BC algorithm in cytoNCA; the redder the color, the higher the ranking, and the yellower the color, the lower the ranking. (d–f) Expression levels of hub genes in GSE93798, GSE115857, and GSE35487. ^∗^*p* < 0.05, ^∗∗^*p* < 0.01, ^∗∗∗^*p* < 0.001, and ^∗∗∗∗^*p* < 0.0001 compared to the model group.

To investigate the interactions between individual DE-FRGs, all DE-FRGs were submitted to the STRING database to generate a PPI network (Figure 4(a)) with the local software Cytoscape (Version 3.9.1). After hiding the solitary DE-FRGs, the PPI network of DE-FRGs was shown, with 17 nodes, 23 edges, and an average node degree of 2.71 (Figure 4(b)). Based on the BC values calculated using the cytoNCA plug-in, the top three important genes were obtained and selected as hub genes, including *JUN*, *IL1B*, and *ATF3* (Figure 4(c)). These selected genes were assessed in subsequent analyses. To verify the accuracy of the genes screened, we tested their expression levels in two external datasets: GSE115857 (55 IgAN, 7 living healthy donors) and GSE35487 (25 IgAN, 6 living healthy donors). The findings demonstrated that JUN and ATF3 were consistently downregulated in GSE115857 and GSE35487 as in GSE93798, whereas IL1B showed no significant change (Figures 4(d), 4(e), and 4(f)). To further investigate the role of these genes in IgAN, we searched the literature and found that JUN, ATF3, and IL1B [18–20] are hub genes in IgAN, further supporting our findings. Collectively, we determined that JUN, IL1B, and ATF3 were the final hub genes for the subsequent analysis.

### 3.5. GSEA of the Hub Genes

**Figure 5:**
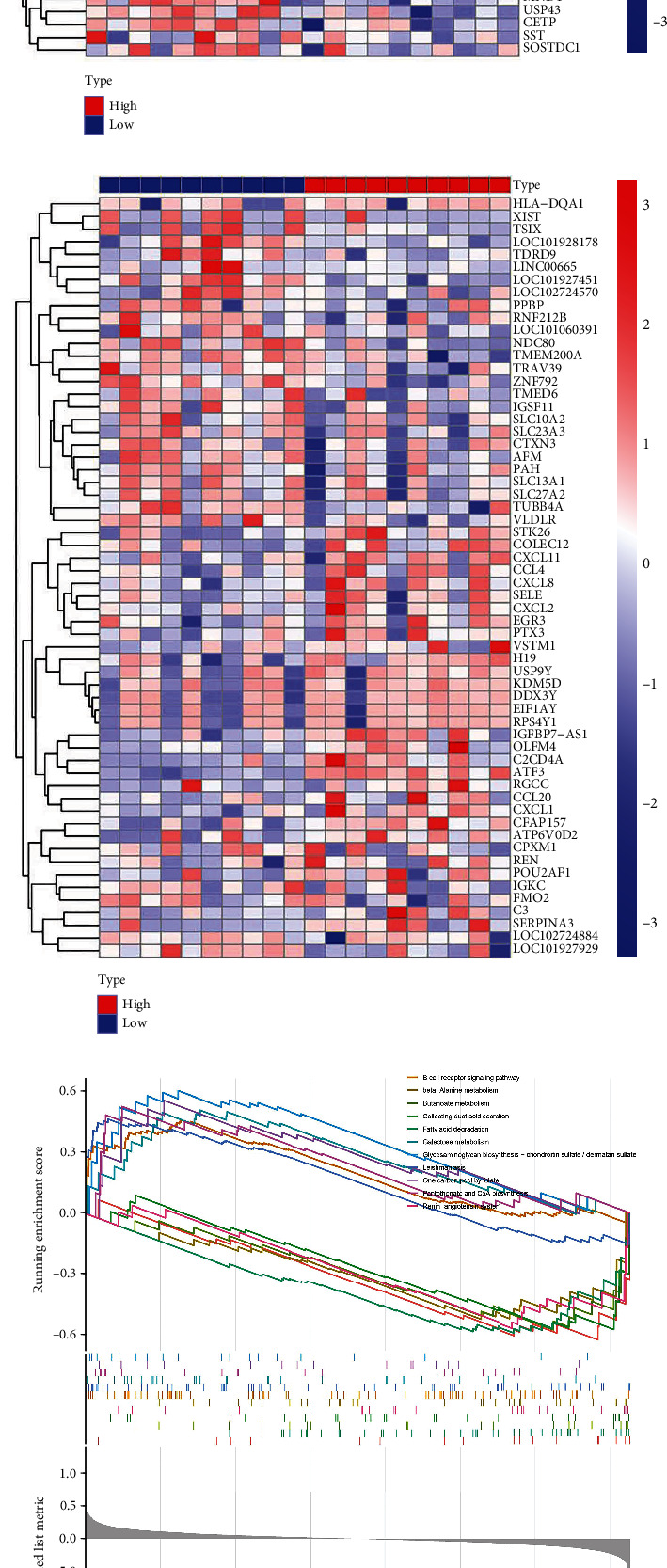
Heat map of genes upregulated and downregulated according to the logFC of (a) JUN, (b) IL1B, and (c) ATF3. Enriched KEGG pathways concerning (d) JUN, (e) IL1B, and (f) ATF3 using the GSEA.

The 20 samples were split into high- and low-expression groups according to the average expression levels of individual hub genes. In Figures 5(a), 5(b), and 5(c), the heat map demonstrated the correlation between differential genes and hub genes. Meanwhile, the KEGG enrichment analysis was used to search for the possible potential mechanism of action of ferroptosis and related signaling pathways in IgAN. Following that, we identified a multitude of KEGG pathways with high enrichment scores using NES values and P screening (Figure 5).

After comprehensive analysis, it was revealed that the signaling pathways associated with JUN expression were mainly glycosaminoglycan biosynthesis (chondroitin sulfate/dermatan sulfate), one carbon pool by folate, pantothenate and CoA biosynthesis, galactose metabolism, leishmaniasis, and B cell receptor signaling pathways; the inhibition pathways were mainly ascorbate and aldarate metabolism, fatty acid degradation, butanoate metabolism, collecting duct acid secretion, renin–angiotensin system, and beta-alanine metabolism (Figure 5(d)). The main signaling pathways associated with IL1B were identified as rheumatoid arthritis, malaria, glycine serine and threonine metabolism, phenylalanine metabolism, African trypanosomiasis, IL-17 signaling pathway, human immunodeficiency virus 1 infection, chemical carcinogenesis-receptor activation, and pathogenic *Escherichia coli* infection; the main inhibited pathways included ECM–receptor interaction, selenocompound metabolism, and taurine and hypotaurine metabolism (Figure 5(e)). As for the ATF3-related pathways, malaria, rheumatoid arthritis, legionellosis, pertussis, NF-kappa B signaling pathway, and TNF signaling pathway were activated, and drug metabolism-cytochrome P450, ascorbate and aldarate metabolism, histidine metabolism, glycine serine and threonine metabolism, arginine biosynthesis and taurine and hypotaurine metabolism were inhibited (Figure 5(f)). As far as our results obtained by GSEA are concerned, the ferroptosis genes may have some connection with IgAN metabolism, immunology, and cytokines, but the specifics still need to be verified by biological experiments.

### 3.6. Immune Landscape Analysis

The previous analyses have shown that the DE-FRGs are closely associated with the immune response. At the same time, it is well recognized that there is an inextricable link between the immune microenvironment and IgAN. Thus, we used the ssGSEA method to investigate the variations in immune microenvironment between the control and IgAN samples and further analyze the association between 28 immune cell species and ferroptosis-associated hub genes.

**Figure 6:**
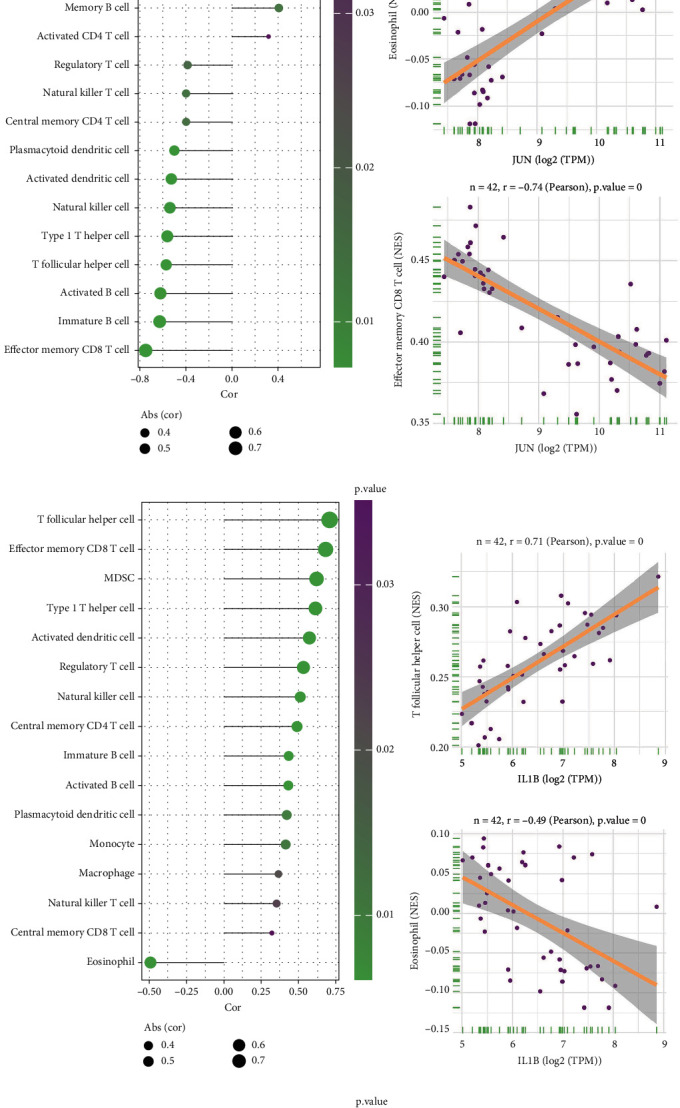
Immune landscape analysis. (a) The ssGSEA algorithm was implemented to explore the changes in the immune microenvironment between IgAN patients and normal samples. Pearson correlation analysis of immune cells with (b) JUN, (d) IL1B, and (f) ATF3. The immune cells with the most positive and negative correlations with (c) JUN, (e) IL1B, and (g) ATF3, respectively. ^∗^*p* < 0.05, ^∗∗^*p* < 0.01, ^∗∗∗^*p* < 0.001, and ^∗∗∗∗^*p* < 0.0001 compared to the model group.

In Figure 6(a), the percentage of some immune cells in the IgAN is significantly less than in the normal sample, such as CD56bright natural killer cell, eosinophil, mast cell, memory B cell, neutrophil and Type 17 T helper cell (all *p* < 0.05); in contrast, the proportion of activated B cell, activated dendritic cell, central memory CD4 T cell, effector memory CD8 T cell, immature B cell, MDSC, monocyte, natural killer cell, natural killer T cell, plasmacytoid dendritic cell, regulatory T cell, T follicular helper cell (Tfh), and Type 1 T helper cell (all *p* < 0.05) was notably higher than that of the control samples.

Moreover, the Pearson correlation analysis indicated a significant relationship between 28 immune cell species and hub genes. As shown in the figure, JUN has strong positive correlations with eosinophil, neutrophil, CD56bright natural killer cell, mast cell, Type 17 T helper cell, memory B cell, and activated CD4 T cell, respectively. Meanwhile, JUN was negatively correlated with regulatory T cell, natural killer T cell, central memory CD4 T cell, plasmacytoid dendritic cell, activated dendritic cell, natural killer cell, Type 1 T helper cell, Tfh, activated B cell, immature B cell, and effector memory CD8 T cell (Figure 6(b)). Furthermore, we presented the pathways with the highest positive and negative correlations (Figure 6(c)). IL1B was positively correlated with Tfh, effector memory CD8 T cell, MDSC, Type 1 T helper cell, activated dendritic cell, regulatory T cell, natural killer cell, central memory CD4 T cell, immature B cell, activated B cell, plasmacytoid dendritic cell, monocyte, macrophage, natural killer T cell, and central memory CD8 T cell and negatively correlated with eosinophil (Figure 6(d)). The most positively and negatively correlated pathways are shown in Figure 6(e). Finally, ATF3 was positively correlated with eosinophil, memory B cell, mast cell, neutrophil, activated CD4 T cell, Type 17 T helper cell, and CD56bright natural killer cell and negatively correlated with MDSC, natural killer T cell, regulatory T cell, Type 1 T helper cell, activated dendritic cell, natural killer cell, plasmacytoid dendritic cell, Tfh, immature B cell, activated B cell, and effector memory CD8 T cell (Figure 6(f)).  Figure 6(g) depicts its most positively and negatively associated pathways. This evidence revealed that the abnormal immune microenvironment of IgAN patients might well be associated with the expression levels of *JUN*, *IL1B*, and *ATF3*.

## 4. Discussion

Ferroptosis is a form of programmed adaptive and cell death that is characterized by the accumulation of lipid peroxidation products and fatal ROS produced by iron metabolism [21]. IgAN is a special type of renal disease in which large amounts of immune deposits, mainly including IgA immune complexes, are present in the glomerular mesangial region, causing functional and structural damage to the kidney [22]. The activation of the nuclear factor-erythroid 2-related factor 2 (NRF2) antioxidant pathway remarkably modified proteinuria and kidney function in IgAN mice, according to an animal study [14]. Another study showed that decreasing of the PPAR*α* pathway reduced fatty acid-binding protein 1 (FABP1) expression levels and affected glutathione peroxidase-4 (GPX4) and acyl-CoA synthetase long-chain family member 4 (ACSL4) expression levels, which could lead to ferroptosis in mesenchymal cells and contribute to the pathogenesis of IgAN [15]. However, the precise mechanism by which ferroptosis plays a role in IgAN needs to be further explored.

For this study, we analyzed gene expression data from the GEO database using bioinformatics to derive a set of 347 DEGs between healthy control and IgAN cohorts. After taking the intersection of DEGs and the FRGs, we acquired 17 overlapping genes. Next, we further explored the biological function of these 17 candidate genes and then selected the core DEGs, including JUN, IL1B, and ATF3, as the final hub genes. Finally, the dataset was divided into high- and low-expression groups based on the average expression values of each hub gene and subjected to ssGSEA. The signaling pathways enriched with respect to the hub genes may provide insights into the mechanism of ferroptosis in the pathogenesis of IgAN. Meanwhile, immune infiltration analysis was performed on the entire dataset and hub genes to explore whether there is a significant association between ferroptosis-associated genes and the immune system in IgAN.

In total, 17 ferroptosis-associated DEGs, including CD44, CDO1, CYBB, IL1B, RRM2, AKR1C1, ATF3, CDKN1A, GDF15, JUN, MGST1, MIOX, MT1G, NR4A1, PDK4, TNFAIP3, and ZFP36, were screened in this study. These genes were sequenced according to BC. Among them, JUN, IL1B, and ATF3 were the top three genes. JUN is a component of the transcription factor cytosolic protein-1 (AP-1). AP-1 mediates multiple BPs such as apoptosis, proliferation, differentiation, and transformation and regulates gene responses to a large number of physiological and pathological stimuli [23, 24]. According to a study by Li et al., lowering the level of phosphorylated c-jun prevented mesangial cells (MCs) from proliferation in mice and thus ameliorated the renal pathological lesions in IgAN [25]. Meanwhile, in vivo and in vitro experiments showed that *Tripterygium wilfordii* Hook F (TwHF) reduced the phosphorylation level of *JUN* in mouse kidneys and human MCs (HMCs), which could potentially inhibit HMC proliferation and the progression of IgAN [26]. TwHF is a vine shrub plant of the genus Leioglossus in the family Wesleyanaceae, which possesses significant anti-inflammatory and immunomodulatory properties and is widely used for a variety of autoimmune-mediated inflammatory diseases. Furthermore, *JUN* may serve as a novel diagnostic marker to help doctors distinguish patients with IgAN. A significant downregulation of JUN in patients with IgAN and significant correlation with proteinuria, glomerular filtration rate (GFR), and serum creatinine levels have been found by evaluating gene expression profiles in patients with IgAN [20]. In addition, *JUN* plays a crucial role in controlling ferroptosis. It was found that *JUN* protein level was significantly modified by O-linked *β*-N-acetylglucosamine (O-GlcNAcylation), thereby inhibiting ferroptosis in human hepatoma cells such as Bel-7402 and SMMC-7721 [27]. In another study, Yang et al. found that c-jun binds to NRF2, regulates downstream heme oxygenase-1 (HO-1), and breaks the vicious cycle between HO-1 activation and lipid peroxidation, effectively inhibiting ferroptosis in osteocytes and deterioration of bone trabeculae [28]. In addition, the overexpression of c-jun also inhibited ferroptosis in Schwann cells (SCs) by suppressing the NRF2/HO-1 pathway. Following c-jun overexpression in animal experiments, prostaglandin-endoperoxide synthase 2 (PTGS2) expression was significantly reduced, while GPX4 and S-100 protein were substantially enhanced, suggesting that c-jun overexpression could inhibit ferroptosis injury to nerve cells and repair the function of the facial nerve [29]. Although there are no reports on the role of *JUN* in the mechanism of ferroptosis in IgAN, our discussion suggests that the effect of ferroptosis on MCs in IgAN may be lessened by employing lower JUN phosphorylation levels and upregulating JUN expression.

IL-1*β* is a significant proinflammatory cytokine. The amount of IgA1 and the degree of IgA1 glycosylation might be controlled by influencing the production of IL-1 and IL-8 [30]. Moreover, IL-1*β* has been a central target for ferroptosis-related diseases, according to several studies [31, 32]. ATF3 is a stress response factor that is a member of the AP-1 family [33]. In addition to regulating the stress response, ATF3 is involved in multiple pathological and physiological processes, including the maintenance of homeostasis, apoptosis, and signaling [34]. ATF3 performs various functions in different mammalian cells and takes on various or even opposing roles depending on the cellular state [33, 34]. ATF3 may play a role in the pathogenesis of IgAN and may be a crucial gene according to a recent study [19]. RNA-seq analysis indicated that the NRF2-ATF3/4-CHAC1 signaling pathway, which includes ChaC glutathione-specific *γ*-glutamyl cyclase 1, may play a crucial role in GSH degradation [35]. Some findings show that knockdown or overexpression of ATF3 could alter the transcriptional levels of many FRGs. In cardiac myocytes, the overexpression of ATF3 inhibited ferroptosis induced by erastin and may be related to the regulation of Fanconi anemia complementation group D2 (FANCD2) [36]. Meanwhile, decreased ATF3 expression could attenuate ionizing radiation (IR)–induced ferroptosis damage in colon cancer cells [37]. In another study, quercetin was found to increase the levels of SLC7A11 and GPX4 expression and subsequently enhance cell viability, by inhibiting ATF3, thereby reducing the risk of acute kidney injury (AKI) [38]. In the discussion, we discovered that ATF3 and IL-1*β* participate in a number of mechanisms that control ferroptosis, but the IgAN mechanism still has to be experimentally validated.

According to our KEGG and GO studies, the underlying process of ferroptosis in IgAN may involve many pathways and sites. Overall, genes were mainly enriched in immune inflammatory pathways, such as NOD-like receptor signaling, IL-17 signaling, and TNF signaling. In accordance with our findings, previous studies have demonstrated these BPs in IgAN [39]. The NOD-like receptor pyrin domain-containing-3 (NLRP3) is a vital member of the NOD-like receptor family, responsible for regulating inflammatory cytokines [40]. NLRP3 has been shown to be involved in the progression of IgAN and holds the potential to be a new therapeutic target [41, 42]. NLRP3 has been extensively reported to be involved in the treatment of IgAN, including probiotics, artemisinin, and osthole [43–45]. Meanwhile, a study by Xie et al. pointed out that ferroptosis-induced inflammatory responses may be triggered in pulmonary artery endothelial cells via the HMGB1/TLR4/NLRP3 signaling pathway [46]. IL-17 is an emerging family of inflammatory cytokines whose signaling pathway can influence downstream genes through activation of NF-*κ*B, MAPK, and transcription factors C/EBP*β* and C/EBP*δ* [47]. In proteomic analysis of lysine 2-hydroxyisobutyrylation (Khib), IgAN was enriched in the IL-17 signaling pathway and phagosome class, which may have an important link to the development of IgAN [48]. Additionally, a bioinformatic study identified the IL-17 signaling pathway as a ferroptosis-related enriched pathway involved in disease progression [49]. Tumor necrosis factor *α* (TNF*α*) is a cellular signaling protein involved in systemic inflammation, mainly through mediating cell survival and cell death–induced signaling and participating in the function of the immune system [50]. A previous study noted that after IgA deposition, TNF-*α* produced by HMCs activated tubular epithelial cells, leading to an inflammatory response in the renal mesangium and promoting the progression of IgAN [51]. Our discoveries contribute to the description of immune and inflammatory pathways, which could enhance our understanding of IgAN mechanisms.

We conducted a GSEA on three key genes, which largely corroborated the KEGG findings, barring minor deviations in infection and metabolism-related pathways. Notably, immune inflammation pathways were significantly enriched. Further, our analysis of immune cell infiltration, via principal component assessment, underscored distinct disparities in immune infiltration between normal and IgAN samples. The differences in mast cells, neutrophils, Tfh, memory B cells, activated B cells, and immature B cells are more meaningful when compared to the published literature. In the observation of specimens from patients with IgA nephropathy, the number of renal tubulointerstitial mast cells showed a significant correlation with the degree of tubulointerstitial fibrosis [52] and may be involved in the inflammatory and fibrotic processes of IgAN [53]. In addition, intraglomerular IgA deposition is capable of recruiting neutrophils to sites of inflammation, further inducing an inflammatory response, and it affects the treatment and prognosis of this IgAN as much as mast cells. The production of abnormally glycosylated IgA1 and the deposition of immune complexes play a crucial role in the pathogenic mechanism of IgAN [54]. B cells are the central hub for producing galactose-deficient IgA1 (Gd-IgA1) and its corresponding antibodies [55]. Additionally, the rise of activated B cells and immature B cells seems to coincide with the onset of IgAN. On this basis, memory B cells are critical for the production of antibody responses upon reexposure to antigens. In IgAN, aberrant immunity to mucosal infections can stimulate memory B cells to produce aberrant IgA, leading to disease pathology. B cells can be divided into several subclasses, and it has been demonstrated that GPX4 plays an essential role in the development and homeostasis of B1 and marginal zone (MZ) B cells [56], which indicates that ferroptosis may regulate the development of disease by affecting B cell. In our study, *JUN* was inversely correlated with immature B cells. JUN has been shown to increase the production of GPX4 [29]. Also, *ATF3* was inversely correlated with activated B cells and immature B cells. However, inhibition of *ATF3* promotes the production of GPX4, which is consistent with our study [38]. Surprisingly, Tfh cells are a specialized class of CD4+ T cells whose abnormality leads to overactivation of B cells and production of excess IgA1 or induces aberrant IgA1 production and formation of depositional complexes in glomeruli, which in turn induces inflammation [57, 58]. B cell lymphoma 6 (Bcl6) has been referred to as a Tfh cell, a key transcription factor for development. It has been shown that *ATF3* is able to influence Bcl6 expression and mediate the differentiation of Tfh cells in the gut [59]. In summary, targeting *ATF3* may have promising therapeutic potential in enhancing the renal immune environment of individuals with IgAN.

In addition, we identified multiple FRGs in IgAN, including *CD44*, *CDO1*, *CYBB*, *IL1B*, *RRM2*, *AKR1C1*, *ATF3*, *CDKN1A*, *GDF15*, *JUN*, *MGST1*, *MIOX*, *MT1G*, *NR4A1*, *PDK4*, *TNFAIP3*, and *ZFP36*. We focused on two genes, JUN and ATF3. These two genes are not only closely associated with ferroptosis but also correlated to the immune microenvironment in the IgAN patients. Needless to say, our study has multiple limitations. To begin with, the results are mainly based on bioinformatic methods, and the samples studied are small and not ethnically diverse, thus requiring larger datasets. Second, mRNA expression levels cannot be equated with protein expression levels, which are based on limited genetic data, which may still bias the final results. Finally, key genes and immune cell infiltration derived from bioinformatic analyses require relevant in vivo and in vitro experiments to validate our conclusions. Therefore, we will continue to focus on these genes and avoid potential sources of bias or confusion. We will also conduct more experiments to further elucidate their relationship with IgAN.

Meanwhile, the results of our experiment hold undeniable significance. Though renal biopsy remains the most authoritative diagnostic tool available [60], its invasive nature carries significant risks. Moreover, the pathogenesis of IgAN has yet to be thoroughly elucidated [61], and the primary therapeutic approaches, encompassing immunosuppression and supportive therapy, have proven unsatisfactory. Under these circumstances, our findings offer novel insights for the examination, diagnosis, and management of IgAN. Initially, the ferroptosis mechanism not only expedites renal fibrosis but can also impact B cell development, a probable crucial factor in the onset and progression of IgAN. Furthermore, *JUN* can be applied for noninvasive detection of IgAN, which has the potential to supplant renal biopsy and minimize avoidable hazards. Finally, *JUN* and *ATF3*, which are essential genes affecting ferroptosis and immune infiltration in IgAN, can help find potential drugs for treating IgAN and improve the renal immune microenvironment. In the future, we plan to use machine learning and deep learning algorithms to identify more potential candidate genes and validate our results through wet experiments, when conditions allow.

## 5. Conclusions

In summary, our study screened hub genes and pathways related to ferroptosis through the bioinformatic approaches, elaborating the potential mechanism of ferroptosis in IgAN, which may provide new clues to explore the specific mechanism of the disease. Further investigation into the relationship between ferroptosis and IgAN is warranted, as it may give novel insights for IgAN diagnosis and therapy.

## Data Availability

The data used to support the results of this paper are available from the GEO database and the FerrDb database.

## Supporting Information

Supporting Information 1 Figure S1: Cross comparability evaluation of microarray data.

Supporting Information 2 Table S1: Ferroptosis genes.

Supporting Information 3 Table S2: Each hub gene's specific enrichment results.
