# Supplementary figures and images for: Ferroptosis-Related Genes in IgA Nephropathy: Screening for Potential Targets of the Mechanism
Source: Int J Genomics. 2024 Aug 14;2024:8851124. doi: 10.1155/2024/8851124 (PMC11338665; doi:10.1155/2024/8851124)

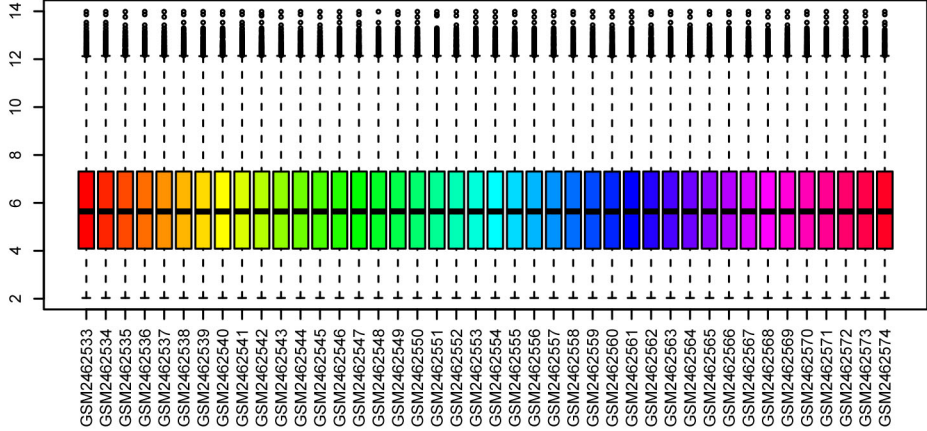

Supplement: Supporting Information 1 — Figure S1: Cross comparability evaluation of microarray data. [file 8851124.f1.pdf]
