# Supplementary material for: Ferroptosis-Related Genes in IgA Nephropathy: Screening for Potential Targets of the Mechanism
Source: Int J Genomics. 2024 Aug 14;2024:8851124. doi: 10.1155/2024/8851124 (PMC11338665; doi:10.1155/2024/8851124)
Supplement: Supporting Information 2 — Table S1: Ferroptosis genes. [file 8851124.f2.pdf]

symbol
RPL8
IREB2
ATP5MC3
CS
EMC2
ACSF2
NOX1
CYBB
NOX3
NOX4
NOX5
DUOX1
DUOX2
G6PD
PGD
VDAC2
TP53
ACSL4
LPCAT3
NRAS
KRAS
HRAS
CARS1
KEAP1
HMOX1
ATG5
ATG7
NCOA4
TF
ALOX5
ALOX12
ALOX12B
ALOX15
ALOX15B
ALOXE3
PHKG2
SAT1
EGFR
MAPK3
MAPK1
ZEB1
DPP4
CDKN2A
PEBP1
SOCS1
CDO1
MYB
SLC1A5
CHAC1
LINC00472

GOT1
BECN1
PRKAA2
PRKAA1
ELAVL1
BAP1
ABCC1
MIR6852
ACVR1B
TGFBR1
IFNG
ANO6
HMGB1
TNFAIP3
ATF3
ATM
YY1AP1
EGLN2
MIOX
TAFAZZIN
MTDH
IDH1
FBXW7
PANX1
DNAJB6
LONP1
CD82
IL1B
POR
CYB5R1
ELOVL5
FADS1
PTEN
IL6
miR-182-5p
miR-378a-3p
CTSB
ATF4
LINC00618
MT1DP
PEX10
AGPAT3
PEX12
CHP1
GPAT4
BRPF1
OSBPL9
INTS2
MMD
CYP4F8
MLLT1

TTPA
GRIA3
EPT1
POM121L12
LIG3
AEBP2
AGPS
CDCA3
PEX2
PEX6
TIMM9
DCAF7
LCE2C
FAR1
PHF21A
SMAD7
LYRM1
AMN
PEX3
MTCH1
SIRT1
ACADSB
PVT1
hsa_circ_0008367
GSK3B
MAPK8
BRD7
SLC25A28
SLC11A2
ZFAS1
SLC38A1
TSC1
TGFB1
SNCA
SIRT3
TFRC
CGAS
STING1
HDDC3
MIR761
MDM2
MDM4
MIR214
DLD
WWTR1
PRKCA
SMPD1
MYCN
IFNA1
IFNA2
IFNA4

IFNA5
IFNA6
IFNA7
IFNA8
IFNA10
IFNA13
IFNA14
IFNA16
IFNA17
IFNA21
SMG9
TLR4
PAQR3
MICU1
TOR2A
MIR375
CircKDM4C
MIR324
QSOX1
CLTRN
KLF2
MIR5096
H19
YTHDC2
DDR2
SLC39A7
TRIM46
ACSL1
KDM5A
CYGB
GSTZ1
ACO1
GJA1
SLC7A11
CIRBP
circPSEN1
YAP1
TRIM26
NDRG1
MIR302A
ASMTL-AS1
FADS2
PIEZO1
LIFR
PTPN6
ADAM23
ARHGEF26-AS1
CPEB1
MIR15A
KDM6B
METTL14

MIB1
KDM5C
CCDC6
MIR539
PTGS2
FTH1
GPX4
AKR1C1
AKR1C2
AKR1C3
RB1
HSPB1
HSF1
NFE2L2
SQSTM1
NQO1
MUC1
MT1G
SLC40A1
CISD1
HSPA5
HELLS
SCD
SRC
STAT3
PML
NFS1
TP63
CDKN1A
MIR137
FH
CISD2
MIR9-1
MIR9-2
MIR9-3
CBS
ISCU
ACSL3
OTUB1
CD44
LINC00336
BRD4
PRDX6
MIR17
SESN2
NF2
ARNTL
HIF1A
JUN
CA9
TMBIM4

PLIN2
AIFM2
LAMP2
ZFP36
PROM2
CHMP5
CHMP6
CAV1
GCH1
PIR
GCLC
HCAR1
SLC16A1
RRM2
NR4A1
PIK3CA
RPTOR
SREBF1
SREBF2
FZD7
P4HB
BCAT2
PLA2G6
MIR424
PARK7
FXN
SUV39H1
ATF2
STK11
FNDC5
CircIL4R
CDH1
NEDD4L
FTMT
BRD2
BRD3
BRDT
DECR1
GLRX5
NCOA3
NR5A2
MTOR
PANX2
RHEBP1
TFAP2A
CP
ARF6
GDF15
ABHD12
TFAM
KDM3B

RNF113A
AHCY
circ-TTBK2
MIR522
IDH2
PPARA
SIAH2
NEDD4
PRDX1
AR
MTF1
COPZ1
NUPR1
USP35
NEAT1
PARP1
PARP2
PARP3
PARP4
PARP6
PARP8
PARP9
PARP10
PARP11
PARP12
PARP14
PARP15
PARP16
PDSS2
OIP5-AS1
MIR190A
CREB1
CREB3
CREB5
MIR130B
BEX1
FABP4
AKT1S1
MLST8
TYRO3
SIRT6
TMSB4X
TMSB4Y
KIF20A
ECH1
circRHOT1
ETV4
MEG8
VCP
circ_0007142
RBMS1

KDM4A
MGST1
circKIF4A
miR-7-5p
circ_0067934
MPC1
CAMKK2
SOX2
SRSF9
MIR4443
MIR27A
MIR670
MEF2C
EZH2
PEDS1
CDC25A
LCN2
TRIB2
DHODH
MIR545
PDK4
CircPVT1
circDTL
PTPN18
FTL
ABCC5
CISD3
FURIN
circRHBG
GALNT14
KLHDC3
LINC01833
circGFRA1
GSTM1
circ0097009
TMEM161B-DT
circEPSTI1
MIR18A
RARRES2
USP11
