# Supplementary material for: Ferroptosis-Related Genes in IgA Nephropathy: Screening for Potential Targets of the Mechanism
Source: Int J Genomics. 2024 Aug 14;2024:8851124. doi: 10.1155/2024/8851124 (PMC11338665; doi:10.1155/2024/8851124)
Supplement: Supporting Information 3 — Table S2: Each hub gene's specific enrichment results. [file 8851124.f3.pdf]

JUN

ID	Descriptic	setSize	enrichment
hsa00053	hsa00053	Ascorbate and aldarate metabolism	17
hsa00071	hsa00071	Fatty acid degradation	41
hsa00650	hsa00650	Butanoate metabolism	22
hsa04966	hsa04966	Collecting duct acid secretion	27
hsa04614	hsa04614	Renin-angiotensin system	22
hsa00410	hsa00410	beta-Alanine metabolism	30
hsa00140	hsa00140	Steroid hormone biosynthesis	47
hsa00830	hsa00830	Retinol metabolism	49
hsa00020	hsa00020	Citrate cycle (TCA cycle)	28
hsa04710	hsa04710	Circadian rhythm	33
hsa00563	hsa00563	Glycosylphosphatidylinositol (GPI)-anchor biosynt	25
hsa04672	hsa04672	Intestinal immune network for IgA production	41
hsa04960	hsa04960	Aldosterone-regulated sodium reabsorption	36
hsa00280	hsa00280	Valine, leucine and isoleucine degradation	46
hsa02010	hsa02010	ABC transporters	44
hsa04940	hsa04940	Type I diabetes mellitus	38
hsa05330	hsa05330	Allograft rejection	33
hsa05332	hsa05332	Graft-versus-host disease	34
hsa05320	hsa05320	Autoimmune thyroid disease	44
hsa00982	hsa00982	Drug metabolism - cytochrome P450	48
hsa00980	hsa00980	Metabolism of xenobiotics by cytochrome P450	52
hsa05323	hsa05323	Rheumatoid arthritis	84
hsa04146	hsa04146	Peroxisome	79
hsa04310	hsa04310	Wnt signaling pathway	162
hsa05170	hsa05170	Human immunodeficiency virus 1 infection	197
hsa04621	hsa04621	NOD-like receptor signaling pathway	164
hsa04062	hsa04062	Chemokine signaling pathway	179
hsa04072	hsa04072	Phospholipase D signaling pathway	142
hsa05167	hsa05167	Kaposi sarcoma-associated herpesvirus infection	183
hsa05202	hsa05202	Transcriptional misregulation in cancer	164
hsa05135	hsa05135	Yersinia infection	130
hsa04670	hsa04670	Leukocyte transendothelial migration	105
hsa04530	hsa04530	Tight junction	158
hsa05161	hsa05161	Hepatitis B	154
hsa04145	hsa04145	Phagosome	136
hsa05414	hsa05414	Dilated cardiomyopathy	93
hsa05152	hsa05152	Tuberculosis	166
hsa04650	hsa04650	Natural killer cell mediated cytotoxicity	112
hsa04512	hsa04512	ECM-receptor interaction	85
hsa05210	hsa05210	Colorectal cancer	85
hsa04668	hsa04668	TNF signaling pathway	109
hsa04061	hsa04061	Viral protein interaction with cytokine and cytol	91
hsa05142	hsa05142	Chagas disease	98
hsa05220	hsa05220	Chronic myeloid leukemia	74
hsa04625	hsa04625	C-type lectin receptor signaling pathway	102
hsa01522	hsa01522	Endocrine resistance	94
hsa04380	hsa04380	Osteoclast differentiation	120
hsa05222	hsa05222	Small cell lung cancer	88
hsa05146	hsa05146	Amoebiasis	100
hsa05144	hsa05144	Malaria	46
hsa05133	hsa05133	Pertussis	71
hsa05214	hsa05214	Glioma	74

hsa04613	hsa04613	Neutrophil extracellular trap formation	102
hsa04662	hsa04662	B cell receptor signaling pathway	78
hsa05140	hsa05140	Leishmaniasis	66
hsa00052	hsa00052	Galactose metabolism	30
hsa00770	hsa00770	Pantothenate and CoA biosynthesis	20
hsa00670	hsa00670	One carbon pool by folate	17
hsa00532	hsa00532	Glycosaminoglycan biosynthesis - chondroitin sul[illegible]	20
hsa04662	hsa04662	B cell receptor signaling pathway	78
hsa05140	hsa05140	Leishmaniasis	66
hsa00052	hsa00052	Galactose metabolism	30
hsa00770	hsa00770	Pantothenate and CoA biosynthesis	20
hsa00670	hsa00670	One carbon pool by folate	17
hsa00532	hsa00532	Glycosaminoglycan biosynthesis - chondroitin sul[illegible]	20
hsa00053	hsa00053	Ascorbate and aldarate metabolism	17
hsa00071	hsa00071	Fatty acid degradation	41
hsa00650	hsa00650	Butanoate metabolism	22
hsa04966	hsa04966	Collecting duct acid secretion	27
hsa04614	hsa04614	Renin-angiotensin system	22
hsa00410	hsa00410	beta-Alanine metabolism	30

NES	pvalue	p.adjust	qvalues	rank	leading_ec
-0.623002474	-1.692530049	0.01454052	0.01454052	0.13994023	1069
-0.589242652	-1.962713797	0.000384911	0.000384911	0.038133871	3851
-0.57380221	-1.665098712	0.013921114	0.013921114	0.13994023	2543
-0.572101291	-1.740936707	0.007159443	0.007159443	0.139165222	533
-0.566209572	-1.643065872	0.016434648	0.016434648	0.13994023	3912
-0.527165784	-1.635111746	0.011750881	0.011750881	0.13994023	4965
-0.519878971	-1.783146962	0.002116606	0.002116606	0.087467621	746
-0.513608453	-1.775768227	0.002305033	0.002305033	0.087467621	3293
-0.511097647	-1.563312784	0.02810853	0.02810853	0.18723649	5772
-0.504702062	-1.605178065	0.015854602	0.015854602	0.13994023	2238
-0.495915457	-1.480702903	0.048898338	0.048898338	0.218973823	3565
-0.495248249	-1.649626973	0.010007698	0.010007698	0.139165222	2614
-0.477466249	-1.552559517	0.025311601	0.025311601	0.175989249	1599
-0.468237744	-1.59892598	0.012162162	0.012162162	0.13994023	2717
-0.465549601	-1.572787197	0.015286378	0.015286378	0.13994023	2396
-0.461186393	-1.512578712	0.029792997	0.029792997	0.187419603	2195
-0.460841729	-1.465682607	0.045436968	0.045436968	0.218973823	1361
-0.457455331	-1.463179066	0.045102594	0.045102594	0.218973823	4558
-0.455765373	-1.539732696	0.019156347	0.019156347	0.144608813	33
-0.431689043	-1.486448055	0.029055224	0.029055224	0.18723649	2066
-0.415321967	-1.453922997	0.037688923	0.037688923	0.211868301	2104
-0.371215154	-1.42330222	0.034689668	0.034689668	0.208303988	2195
-0.363401854	-1.377984533	0.046472248	0.046472248	0.218973823	2741
-0.328065242	-1.391749997	0.017876687	0.017876687	0.13994023	5108
0.284318979	1.269412871	0.043951165	0.043951165	0.218973823	3204
0.295640356	1.285813796	0.043237251	0.043237251	0.218973823	3252
0.298545019	1.3144682	0.032984177	0.032984177	0.202668992	3176
0.304044874	1.29362174	0.04839779	0.04839779	0.218973823	3179
0.31518797	1.393392448	0.011479029	0.011479029	0.13994023	4703
0.31538887	1.371705019	0.017960089	0.017960089	0.13994023	3288
0.321881327	1.351363959	0.028552603	0.028552603	0.18723649	3584
0.325088033	1.321350799	0.044357469	0.044357469	0.218973823	2827
0.329124842	1.423699427	0.009521701	0.009521701	0.139165222	2430
0.330222565	1.422255438	0.009745293	0.009745293	0.139165222	3176
0.331004773	1.40023516	0.015734266	0.015734266	0.13994023	993
0.339417425	1.353559699	0.041290323	0.041290323	0.218973823	4157
0.344682614	1.503787025	0.002648422	0.002648422	0.087467621	2160
0.345281883	1.421564683	0.018008245	0.018008245	0.13994023	1925
0.350053026	1.375161151	0.037355088	0.037355088	0.211868301	4050
0.350131821	1.375470695	0.037355088	0.037355088	0.211868301	3131
0.352150097	1.442838659	0.013612792	0.013612792	0.13994023	3176
0.35681131	1.418526836	0.023380523	0.023380523	0.1715939	1791
0.364112512	1.465390922	0.013936535	0.013936535	0.13994023	3490
0.37711098	1.444610281	0.024307036	0.024307036	0.173572293	3493
0.379094614	1.535679055	0.006927906	0.006927906	0.139165222	3480
0.384043984	1.534783412	0.007735281	0.007735281	0.139165222	3288
0.385247774	1.600298786	0.002391824	0.002391824	0.087467621	3176
0.39051811	1.543691811	0.007740271	0.007740271	0.139165222	3176
0.402066383	1.623134728	0.003010105	0.003010105	0.088366836	2048
0.409933877	1.435574967	0.044587308	0.044587308	0.218973823	1875
0.411633826	1.561790754	0.00923143	0.00923143	0.139165222	2447
0.416545763	1.595674281	0.005970149	0.005970149	0.139165222	3493

0.428494016	1.735791704	0.000432994	0.000432994	0.038133871	3182
0.458304673	1.775212602	0.000428357	0.000428357	0.038133871	3176
0.462815564	1.731039176	0.001928434	0.001928434	0.087467621	755
0.506621348	1.612255423	0.017973856	0.017973856	0.13994023	3817
0.524916597	1.506518005	0.047697708	0.047697708	0.218973823	1224
0.554613496	1.524273399	0.048513625	0.048513625	0.218973823	2617
0.601192132	1.725429862	0.009085278	0.009085278	0.139165222	3090
0.458304673	1.775212602	0.000428357	0.000428357	0.038133871	3176
0.462815564	1.731039176	0.001928434	0.001928434	0.087467621	755
0.506621348	1.612255423	0.017973856	0.017973856	0.13994023	3817
0.524916597	1.506518005	0.047697708	0.047697708	0.218973823	1224
0.554613496	1.524273399	0.048513625	0.048513625	0.218973823	2617
0.601192132	1.725429862	0.009085278	0.009085278	0.139165222	3090
-0.623002474	-1.692530049	0.01454052	0.01454052	0.13994023	1069
-0.589242652	-1.962713797	0.000384911	0.000384911	0.038133871	3851
-0.57380221	-1.665098712	0.013921114	0.013921114	0.13994023	2543
-0.572101291	-1.740936707	0.007159443	0.007159443	0.139165222	533
-0.566209572	-1.643065872	0.016434648	0.016434648	0.13994023	3912
-0.527165784	-1.635111746	0.011750881	0.011750881	0.13994023	4965

core_enrichment
tags=53%, 219/217/55586/223/7358/9104/501/54576/54578
tags=54%, 131/23305/10455/3033/1632/219/217/124/1374/128/223/2639/130/38/36/127/33/34/501
tags=36%, 622/38/3157/54988/5019/39/1962/6296
tags=33%, 10723/90423/23545/6521/760/245973/127124/50617/245972
tags=50%, 57486/4311/10159/5550/2028/7064/290/3816/59272/1359/5972
tags=50%, 2572/1806/1807/4329/219/217/55748/223/51380/314/23417/501/26275/8639/1962
tags=17%, 79154/1645/54576/79644/1551/3291/3294/54578
tags=35%, 124/51109/1555/10170/56603/128/9227/130/127/53630/8854/8694/8228/54576/1551/125
tags=61%, 4967/1743/5162/50/5161/5160/3417/6390/47/48/6392/6391/5091/8803/4190/1737/8801
tags=27%, 8553/5564/26224/6095/1628/4783/406/1407/6097
tags=40%, 5277/23556/54965/2822/9488/5279/51227/5281/80235/5283
tags=39%, 3113/3109/10803/3115/3567/3569/6370/29851/5284/3676/3112/56477/608/6387/3119/31
tags=19%, 6337/53828/3667/2810/3758/3479/3291
tags=52%, 3033/3155/11112/4329/219/217/64902/594/56922/223/64087/38/36/5095/3157/549/8469
tags=27%, 10060/10257/5825/6833/23461/64240/5826/1244/22/23460/1672/10351
tags=45%, 3002/1363/2572/3105/3107/3113/3109/3115/5799/3135/3553/3552/3382/3112/3458/3119
tags=12%, 3112/3458/3119/3117
tags=44%, 3812/3107/3113/3109/3115/3135/3802/3569/3804/3553/3552/3112/3458/3119/3117
tags=27%, 3105/3440/3107/3113/3109/3115/3446/3567/3135/3112/3119/3117
tags=21%, 130/2327/4129/127/2940/2329/2328/54576/125/54578
tags=19%, 3290/130/127/22977/2940/1645/54576/2052/125/54578
tags=23%, 3553/3552/4314/90423/6372/23545/3112/7042/3458/1513/2920/245973/127124/2921/638
tags=27%, 8309/5189/5264/2053/55711/26061/92960/8504/847/5825/5824/84188/283927/1891/2341
tags=40%, 6907/64840/81839/7091/8945/440193/54894/5881/5567/1454/6885/2535/8312/1501/1487
tags=23%, 2353/54331/3725/8772/25939/810/3654/7097/7124/2787/5608/7852/57144/2775/3845/10
tags=25%, 1536/3725/84168/820/8772/115362/64127/56919/7124/3576/2635/55669/2634/1670/2919
tags=25%, 3579/5473/5196/3055/54331/6369/2268/6364/1230/6355/6368/10235/157/2787/6357/257
tags=22%, 3579/8525/2207/5156/5154/5321/9162/10554/25759/185/553/3576/8527/5338/23566/384
tags=34%, 2353/3055/54331/3725/1230/8772/5743/1026/810/1827/595/2787/5608/3576/7311/3845/
tags=29%, 3684/7850/7704/1051/1026/5154/3248/1848/10912/2119/4211/3576/904/1670/4094/2120
tags=26%, 2353/3725/653857/2335/3654/3678/60/7124/5608/10093/3576/7454/10095/10454/9475/7
tags=25%, 4688/1536/3684/4267/3689/3683/4633/60/83700/6494/7852/87/81/83593/9475/5880/235
tags=22%, 3725/154796/912/653857/3059/79778/5521/595/8777/4633/60/83700/7074/10093/87/745
tags=26%, 2353/6554/3725/7043/8772/1026/3654/1960/7097/7124/3339/5608/114609/3576/3845/10
tags=14%, 2215/4688/1536/3684/7058/4481/4360/1780/9902/1520/3689/7097/4074/8685/3678/5160
tags=37%, 7043/6445/7134/4634/3678/4633/60/3908/7124/5350/55799/3674/8516/153/779/1756/78
tags=21%, 2215/26253/3684/2207/4360/820/7043/4843/8772/1051/3687/3587/9902/1520/810/3654/
tags=18%, 2215/7305/2207/5777/3689/3683/7124/3811/3824/80329/25759/3384/3845/5551/6464/39
tags=36%, 7058/2335/1284/3914/3910/1278/3678/3908/3339/3674/8516/7148/960/1282/6385/3655/
tags=25%, 2353/3725/7043/1026/374/595/10912/3845/5898/7157/5880/3265/581/332/1647/1643/10
tags=31%, 2353/197259/3726/3725/6364/8809/8772/1051/5743/64127/9530/7124/1326/5608/2919/1
tags=21%, 3579/5473/5196/8807/6369/6364/8809/1230/6355/3587/6368/7124/6357/3576/7852/2919
tags=30%, 2353/5054/3725/7043/4843/8772/3654/5521/7097/714/7124/3576/2775/915/3592/7099/8
tags=35%, 7043/1026/595/10912/25759/3845/3066/6464/7157/3265/581/1871/4790/25/1647/1643/1
tags=30%, 26253/2207/3725/5743/810/1960/7124/51266/3845/3592/6361/3265/841/4790/10379/834
tags=30%, 2353/3725/1026/595/638/25759/4855/3714/4854/3845/6464/1031/10498/7157/3265/581/
tags=30%, 2215/2353/4688/10288/7305/11027/3726/11025/3725/11024/11006/7124/695/5608/54/10
tags=35%, 4843/5743/1026/2335/1284/3914/595/3910/3908/10912/3674/5915/1282/7186/7157/7187
tags=26%, 3684/7850/912/7043/4843/2335/1284/3914/3689/3910/1278/384/7097/3908/7124/3576/8
tags=28%, 7058/3043/7043/3689/3683/2995/7097/7124/3576/2994/3592/958/7099
tags=25%, 2353/3684/3725/4843/810/3654/3689/714/3678/7124/725/114609/3576/3592/7099/4790/
tags=34%, 1026/5156/5154/810/595/57172/10912/25759/3845/6464/7157/3265/581/1871/1647/1643

tags=31%, 2215/4688/1536/3684/2357/820/3689/3683/7097/60/3674/2359/2358/3066/728/1511/588
tags=35%, 2353/10288/11027/11025/3725/11024/5777/11006/695/27071/3636/933/3845/971/5880/3
tags=21%, 2215/2353/4688/1536/3684/3725/7043/4843/5777/5743/3654/3689/7097/7124
tags=47%, 3099/5236/3098/7360/92579/2717/2683/2645/8972/3906/2592/130589/2595/57016
tags=30%, 8876/60490/80025/8875/587/80347
tags=35%, 7298/10841/123263/10797/275/6472
tags=50%, 54480/50515/51363/166012/55454/55790/64132/22856/26229/79586
tags=35%, 2353/10288/11027/11025/3725/11024/5777/11006/695/27071/3636/933/3845/971/5880/3
tags=21%, 2215/2353/4688/1536/3684/3725/7043/4843/5777/5743/3654/3689/7097/7124
tags=47%, 3099/5236/3098/7360/92579/2717/2683/2645/8972/3906/2592/130589/2595/57016
tags=30%, 8876/60490/80025/8875/587/80347
tags=35%, 7298/10841/123263/10797/275/6472
tags=50%, 54480/50515/51363/166012/55454/55790/64132/22856/26229/79586
tags=53%, 219/217/55586/223/7358/9104/501/54576/54578
tags=54%, 131/23305/10455/3033/1632/219/217/124/1374/128/223/2639/130/38/36/127/33/34/501
tags=36%, 622/38/3157/54988/5019/39/1962/6296
tags=33%, 10723/90423/23545/6521/760/245973/127124/50617/245972
tags=50%, 57486/4311/10159/5550/2028/7064/290/3816/59272/1359/5972
tags=50%, 2572/1806/1807/4329/219/217/55748/223/51380/314/23417/501/26275/8639/1962

./39/1962/125

5/54578

.17

)3/34/27034/5019/501/26275/39/1962

)/3117

37/50617/245972/3119/3117
.7/8799/5826/4358/11001/1962/83594
7/1452/79718/11211/85409/56998/894/7976/5532/23401/4088/4919/11197/7482/5467/5601/2239/284
)454/1174/915/7186/200316/5880/5606/3265/7099/637/841/3456/5829/27350/2923/581/4790/9582/5
)/10454/81858/7186/9447/7187/7099/841/3456/118429/837/4790/79792/10379/22900/9140/834/1005
759/7074/3576/7852/7454/3845/2919/9475/6464/5880/6361/56288/3265/5829/4790/51764/114/1236/
15/5898/6464/3630/3265/114/160851/183/6237/5336/5900/10000/9266/112/208/255189
/2919/7186/7157/7187/3265/637/841/3456/581/1871/4790/51764/10379/3455/57580/1870/4792/5336
)/3066/2313/1031/7157/958/8900/5371/4914/4299/7030/581/6688/4790/8861/929/1647/1643/4298/8
7186/3937/5880/5606/7099/3456/5829/4790/834/10451/6196/4792/1398/10000/208/3551/3586/29108
562/7414/1003/5829/90952/10451/4313/9074/4318/5336
54/3308/81/4629/10095/9475/23513/2017/57530/23562/56288/27134/4628/50855/91862/51762/15356
)454/7157/8900/5606/7187/3265/7099/637/841/3456/581/1871/4790/332/64764/1643/1870/4792/431
)6/60/9296/6441
35/3655/114/183/7168/1605/3696/7171/112/488/784/3680/59284/782/59283/7169/108/6443
/3689/64127/7097/51606/7124/4261/114609/10312/1509/3592/7099/637/841/3456/581/4790/7096/15
)37/5880/3265/637/3456
/961/3915/1605/3696/3911/1277/7143/7448/3680/51206/2815/3913/7059/1288/2811
)297/51426/5900/10000/208
.0454/7186/5606/7187/841/3456/4790/10059/64764/4792/4323/4318/8986/7133/1388/10000/843/208
)/57007/6361/3561
341/3456/4790/10333/4792/10000/208/3551/917/3586/718/5518/2767
.870/4792/1398/51426/1029/10000/208/3551/4193/6654
1/6237/4792/5336/5971/10000/338339/4791/6773/208/3551/8844/4193/808/3586/29108
/1871/114/4313/1870/4318/8202/1029/54567/10000/112/208/4193
)454/7186/3937/3456/6688/4790/54209/10379/8651/3455/10326/1436/4792/5336/5971/10000/4791/5
7/581/1871/4790/3655/3915/1647/1643/1870/4792/51426/4149/10000/3911/208/3551
37/1282/81/2919/1511/3592/7414/5052/7099/4790

/834/929
3/1870/5336/51426/1029/10000/208/4193/808/6654

30/10533/7099/837/4790/55869/27180/79792/1183/834/6404/5336/10000/208/291/83933
3265/4790/10451/974/4792/3635/5336/4067/10000/4794/208/3551

3265/4790/10451/974/4792/3635/5336/4067/10000/4794/208/3551

./39/1962/125

4654/817/22943/81029/5602/27130/8607/7855/1488/5530/4775/4041/23236/1460/6423/27121/8313/
51764/545/4792/56924/5336/1398/7133/10000/208/8450/3551/917
59/3455/51393/4792/91662/6773/3551/114769/260434/10392
/10451/57580/4792/5336/1398/4067/10000/6773/112/208/3551

5/4067/10000/6773/208/3551/808/718/3572/8678/836/7538/8717/3459/9976/7314/51806/2793/2353
3938/1050/30012/1436/4318/6495/51426/4149/2521/8148/3398/4066/2322/4193
3/63916

52/55114/123720
18/1388/10000/843/6773/208/3551

594/11151

3/3551

55423/6773/208/3551

5534/4773/57216/164284/4089/25805/10023/51701/5176/50964/166336/64321/7474/8324/6422/8323

3/2033/3709/5609/2786/5155/3661/5600/2791/5603/7297/7422

/8061

ATF3

ID	Descriptic	setSize	enrichment	NES	pvalue	p.adjust
hsa05144	hsa05144	Malaria	46	0.704609503	2.377857081	0.000206398
hsa05323	hsa05323	Rheumatoic	84	0.689997837	2.610199	0.000204332
hsa05134	hsa05134	Legionellc	53	0.67309868	2.340625445	0.000204541
hsa05133	hsa05133	Pertussis	71	0.620906001	2.28946612	0.000203707
hsa04064	hsa04064	NF-kappa E	98	0.611086128	2.37783524	0.000202799
hsa04668	hsa04668	TNF signal	109	0.606574096	2.40044066	0.000201207
hsa05120	hsa05120	Epithelial	69	0.606177822	2.231087361	0.000202306
hsa05150	hsa05150	Staphylocc	72	0.593820235	2.193036761	0.000204123
hsa05330	hsa05330	Allograft	33	0.591552801	1.859328687	0.00165221
hsa05332	hsa05332	Graft-vers	34	0.591104773	1.872576431	0.001444788
hsa04966	hsa04966	Collecting	27	0.590351494	1.773938994	0.003516029
hsa05322	hsa05322	Systemic l	44	0.58844802	1.970737648	0.000620091
hsa05143	hsa05143	African tr	34	0.586974001	1.859490449	0.001651187
hsa05340	hsa05340	Primary in	35	0.579722253	1.845191196	0.001451078
hsa04657	hsa04657	IL-17 sigr	90	0.57922431	2.216653568	0.000204248
hsa00100	hsa00100	Steroid bi	19	0.578212343	1.591690631	0.026326615
hsa04061	hsa04061	Viral prot	91	0.57531156	2.203862368	0.000204248
hsa04940	hsa04940	Type I dia	38	0.563873999	1.829066378	0.001652893
hsa05142	hsa05142	Chagas dis	98	0.563043386	2.190893141	0.000202799
hsa04610	hsa04610	Complement	80	0.562849186	2.109329201	0.00020555
hsa05146	hsa05146	Amoebiasis	100	0.541632401	2.114654381	0.000203046
hsa04620	hsa04620	Toll-like	93	0.540205999	2.082641226	0.000202184
hsa05416	hsa05416	Viral myoc	55	0.53360125	1.868080229	0.001233299
hsa03050	hsa03050	Proteasome	41	0.529848102	1.746341574	0.003320191
hsa05310	hsa05310	Asthma	26	0.512588547	1.525848694	0.033236994
hsa04216	hsa04216	Ferroptosi	37	0.508747882	1.643024565	0.010356255
hsa04672	hsa04672	Intestinal	41	0.503523025	1.659575995	0.007262918
hsa04612	hsa04612	Antigen pr	63	0.502037935	1.810870384	0.001229508
hsa05171	hsa05171	Coronaviru	171	0.497957508	2.095277502	0.000201369
hsa04640	hsa04640	Hematopoie	89	0.494356888	1.890071283	0.000406091
hsa04613	hsa04613	Neutrophil	102	0.483487091	1.894813705	0.000404204
hsa04145	hsa04145	Phagosome	136	0.483039773	1.971534535	0.000202265
hsa04215	hsa04215	Apoptosis	29	0.481862453	1.471985774	0.048624043
hsa04062	hsa04062	Chemokine	179	0.477146475	2.018266787	0.000201898
hsa05320	hsa05320	Autoimmune	44	0.477103	1.597838403	0.011368334
hsa05110	hsa05110	Vibrio chc	50	0.474561105	1.628373508	0.007846376
hsa05219	hsa05219	Bladder ca	41	0.472025371	1.555761974	0.017846026
hsa04623	hsa04623	Cytosolic	54	0.471314699	1.644483139	0.006961507
hsa05140	hsa05140	Leishmania	66	0.468749097	1.702616565	0.002661753
hsa05167	hsa05167	Kaposi sar	183	0.456752106	1.935500211	0.000203211
hsa05145	hsa05145	Toxoplasmc	106	0.455170354	1.793543444	0.000403959
hsa04650	hsa04650	Natural ki	112	0.452372253	1.79304193	0.000405022
hsa05164	hsa05164	Influenza	151	0.450444613	1.866959273	0.000202224
hsa04933	hsa04933	AGE-RAGE s	99	0.449377917	1.753890622	0.000403388
hsa04936	hsa04936	Alcoholic	133	0.447841209	1.822416958	0.000202799
hsa04962	hsa04962	Vasopressi	44	0.447341501	1.498165867	0.029557668
hsa04380	hsa04380	Osteoclast	120	0.446271645	1.790460991	0.000200682
hsa05321	hsa05321	Inflammatc	61	0.444499735	1.594981143	0.009399264
hsa05235	hsa05235	PD-L1 expr	86	0.443694437	1.688691431	0.001013377
hsa04662	hsa04662	B cell rec	78	0.442356298	1.650786035	0.003483607
hsa05152	hsa05152	Tuberculos	166	0.440438693	1.84608226	0.000200682
hsa04920	hsa04920	Adipocytok	65	0.43224552	1.565371556	0.01029654

hsa05221	hsa05221	Acute myel	65	0.431557911	1.562881386	0.01029654
hsa04210	hsa04210	Apoptosis	129	0.429022103	1.738348434	0.000202429
hsa05169	hsa05169	Epstein-Ba	187	0.426231437	1.812984289	0.000202429
hsa05202	hsa05202	Transcript	164	0.410968986	1.718491114	0.000606673
hsa04660	hsa04660	T cell rec	101	0.409366715	1.601487879	0.003437121
hsa04658	hsa04658	Th1 and Th	87	0.403578118	1.538474697	0.009953281
hsa04621	hsa04621	NOD-like r	164	0.4011475	1.677421992	0.000606673
hsa04625	hsa04625	C-type lec	102	0.399057258	1.563928335	0.005254648
hsa04659	hsa04659	Th17 cell	103	0.39490003	1.549901315	0.005656566
hsa05203	hsa05203	Viral carc	156	0.388361446	1.61386446	0.001013993
hsa04611	hsa04611	Platelet a	113	0.385672957	1.530346898	0.00811359
hsa05135	hsa05135	Yersinia i	130	0.385201229	1.561091417	0.004060914
hsa04514	hsa04514	Cell adhes	143	0.38231862	1.571623433	0.002227171
hsa04512	hsa04512	ECM-recept	85	0.380135081	1.443138539	0.025963489
hsa05100	hsa05100	Bacterial	67	0.379712647	1.386317361	0.047171738
hsa05161	hsa05161	Hepatitis	154	0.376456263	1.562812351	0.002631579
hsa04142	hsa04142	Lysosome	129	0.374134492	1.515950117	0.006072874
hsa04066	hsa04066	HIF-1 sigr	103	0.368565607	1.446544128	0.021010101
hsa04630	hsa04630	JAK-STAT s	155	0.367270975	1.52527802	0.003442689
hsa00190	hsa00190	Oxidative	98	0.365579794	1.422530277	0.02494423
hsa05222	hsa05222	Small cell	88	0.357086471	1.362717209	0.04640749
hsa04510	hsa04510	Focal adhe	196	0.327756482	1.403037336	0.011140369
hsa01200	hsa01200	Carbon met	107	-0.360482465	-1.414784939	0.0257222
hsa04976	hsa04976	Bile secre	75	-0.386559306	-1.432877178	0.028638682
hsa04260	hsa04260	Cardiac mu	78	-0.39414616	-1.475338172	0.019133151
hsa00010	hsa00010	Glycolysis	62	-0.400825183	-1.433652795	0.036153996
hsa04146	hsa04146	Peroxisome	79	-0.415942818	-1.560587082	0.009929907
hsa00310	hsa00310	Lysine deg	60	-0.44395264	-1.578711582	0.010735897
hsa05204	hsa05204	Chemical c	46	-0.452760347	-1.529749382	0.020360675
hsa00380	hsa00380	Tryptophan	42	-0.473875434	-1.572084748	0.014986376
hsa01230	hsa01230	Biosynthes	65	-0.47678212	-1.723847819	0.002331908
hsa00980	hsa00980	Metabolism	52	-0.478591481	-1.653695123	0.004502741
hsa00330	hsa00330	Arginine a	46	-0.495867511	-1.675396321	0.005429513
hsa00280	hsa00280	Valine, le	46	-0.532983321	-1.800800162	0.00155129
hsa00770	hsa00770	Pantothena	20	-0.540715567	-1.520856366	0.041868579
hsa00640	hsa00640	Propanoate	30	-0.551914319	-1.713166976	0.005974176
hsa00410	hsa00410	beta-Alani	30	-0.562511111	-1.746059898	0.004817884
hsa00670	hsa00670	One carbon	17	-0.5688859	-1.539979551	0.03962704
hsa00630	hsa00630	Glyoxylate	27	-0.574838539	-1.743798996	0.007354364
hsa04964	hsa04964	Proximal t	22	-0.584558563	-1.685016408	0.012521672
hsa00650	hsa00650	Butanoate	22	-0.586037222	-1.689278707	0.012521672
hsa00250	hsa00250	Alanine, a	35	-0.608347496	-1.950763097	0.000579374
hsa00982	hsa00982	Drug metab	48	-0.610492526	-2.07844666	0.000195236
hsa00053	hsa00053	Ascorbate	17	-0.613313331	-1.660245029	0.015928516
hsa00340	hsa00340	Histidine	22	-0.61858401	-1.783096293	0.005393951
hsa00260	hsa00260	Glycine, s	35	-0.627967573	-2.013677997	0.000193125
hsa00220	hsa00220	Arginine b	20	-0.637356632	-1.792676132	0.0048459
hsa00430	hsa00430	Taurine an	14	-0.703362077	-1.80566144	0.005093046

qvalues	rank	leading_ec	core_enrichment	
0.000206398	0.001754804	2637	tags=50%,	3576/6401/3383/6347/948/7124/3458/3683/
0.000204332	0.001754804	696	tags=29%,	3117/3576/2919/245972/6364/2920/6372/33
0.000204541	0.001754804	2101	tags=38%,	3576/2919/2920/718/2921/7124/3684/3689/
0.000203707	0.001754804	2272	tags=38%,	3576/718/6372/712/713/3394/7124/3684/36
0.000202799	0.001754804	1973	tags=34%,	3576/2919/6351/2920/7128/3383/597/330/2
0.000201207	0.001754804	1680	tags=30%,	2919/6364/2920/6401/7128/6372/3383/6347
0.000202306	0.001754804	1587	tags=28%,	3576/2919/245972/2920/6352/2921/1839/47
0.000204123	0.001754804	1380	tags=29%,	3117/718/3383/712/2266/713/3684/1675/71
0.00165221	0.008119472	2514	tags=33%,	3117/7124/3458/3002/942/3119/5551/3592/
0.001444788	0.007823527	1125	tags=24%,	3117/7124/3458/3002/942/3119/3569/5551
0.003516029	0.014393101	3375	tags=52%,	245972/23545/9114/1188/245973/10312/529
0.000620091	0.003916364	2645	tags=41%,	3117/718/712/713/7124/3458/942/3119/714
0.001651187	0.008119472	3229	tags=47%,	6401/3383/7124/3458/7064/7412/169355/35
0.001451078	0.007823527	3271	tags=40%,	925/3575/915/695/6890/3561/84876/8625/7
0.000204248	0.001754804	1216	tags=22%,	3576/2919/6364/2920/7128/6372/6347/8061
0.026326615	0.06395129	2728	tags=37%,	1591/1717/6713/8435/1594/3988/10682
0.000204248	0.001754804	1773	tags=33%,	3576/2919/6351/6373/6364/2920/6372/6347
0.001652893	0.008119472	2290	tags=29%,	3117/7124/3458/3002/942/3119/5551/3592/
0.000202799	0.001754804	1457	tags=23%,	3576/718/5054/712/6347/713/6352/917/712
0.00020555	0.001754804	2917	tags=44%,	718/5054/712/2266/713/5265/7056/2244/22
0.000203046	0.001754804	2101	tags=29%,	3576/2919/2920/2921/7124/3684/10319/345
0.000202184	0.001754804	1758	tags=26%,	3576/6351/6373/6352/1326/7124/6696/929/
0.001233299	0.006990359	2640	tags=31%,	3117/3383/3683/5880/3689/942/3119/637/5
0.003320191	0.014393101	4638	tags=51%,	3458/11047/5691/5699/5718/5714/5696/568
0.033236994	0.078160905	1401	tags=19%,	3117/7124/2207/3119/2205
0.010356255	0.030122072	4089	tags=46%,	3162/55240/2180/2495/10162/6520/23516/1
0.007262918	0.023907376	2514	tags=34%,	3117/3600/942/2826/3119/3569/3601/4055/
0.001229508	0.006990359	824	tags=16%,	3117/925/7124/3458/3306/10437/3823/6890
0.000201369	0.001754804	1970	tags=25%,	6192/3576/718/712/6347/2266/713/2244/22
0.000406091	0.002895728	1560	tags=22%,	3117/925/948/917/7124/3684/912/3575/929
0.000404204	0.002895728	3229	tags=34%,	718/2266/2244/2243/3684/3683/5880/3689/
0.000202265	0.001754804	1587	tags=22%,	3117/81035/245972/718/948/3684/3689/929
0.048624043	0.107484727	3430	tags=38%,	330/7132/5366/637/4804/79444/836/317/57
0.000201898	0.001754804	2766	tags=30%,	3576/2919/6351/6373/6364/2920/6372/6347
0.011368334	0.031810129	1125	tags=14%,	3117/3002/7252/942/3119/5551
0.007846376	0.025137132	4886	tags=48%,	245972/23545/9114/109/245973/10312/529/
0.017846026	0.046963227	1458	tags=20%,	3576/1026/1839/4312/4609/5605/1871/1890
0.006961507	0.023316052	1993	tags=28%,	6351/6352/4792/90865/3569/4790/3627/106
0.002661753	0.012007907	1880	tags=26%,	3117/718/7124/3684/3458/3689/4792/7097/
0.000203211	0.001754804	2138	tags=21%,	3576/2919/2920/718/3383/1026/2921/7538/
0.000403959	0.002895728	2766	tags=30%,	3117/330/7124/10319/3587/3458/7132/3306
0.000405022	0.002895728	3384	tags=33%,	3383/7124/3458/399694/3683/5880/7409/30
0.000202224	0.001754804	2198	tags=24%,	3117/3576/3383/6347/9021/6352/7124/3458
0.000403388	0.002895728	2123	tags=24%,	3576/6401/5054/3383/6347/7056/7124/1958
0.000202799	0.001754804	2101	tags=20%,	3576/2919/2920/718/712/713/2921/7124/71
0.029557668	0.070255918	4864	tags=39%,	359/396/109/112/5568/84699/6810/360/586
0.000200682	0.001754804	1973	tags=24%,	9021/8061/7124/10288/2354/3458/7132/54/
0.009399264	0.029263833	1773	tags=25%,	3117/7124/3458/6775/8807/30009/7097/311
0.001013377	0.006057996	3384	tags=37%,	55509/917/3458/4794/116071/10125/915/47
0.003483607	0.014393101	3332	tags=32%,	10288/118788/5880/7409/4794/4792/695/68
0.000200682	0.001754804	2272	tags=24%,	3117/245972/718/1051/7124/3684/3587/368
0.01029654	0.030122072	577	tags=15%,	948/9021/7124/7132/4794/23205/4792/9257

0.01029654	0.030122072	2123	tags=26%,	597/5292/3684/1848/929/8900/6776/6198/1
0.000202429	0.001754804	2197	tags=29%,	597/330/7124/7132/3002/1522/5366/4792/8
0.000202429	0.001754804	1758	tags=20%,	3117/7128/3383/1026/917/864/7124/3683/4
0.000606673	0.003916364	2747	tags=24%,	3576/1026/597/330/1051/8013/3684/3002/8
0.003437121	0.014393101	3271	tags=25%,	925/917/1326/7124/3458/7409/4794/10125/
0.009953281	0.030122072	2077	tags=22%,	3117/917/864/3458/6775/4794/30009/915/4
0.000606673	0.003916364	3284	tags=26%,	3576/2919/2920/7128/6347/6352/330/2921/
0.005254648	0.019358197	3052	tags=25%,	1960/1959/7124/602/3659/4792/2207/4046/
0.005656566	0.019847599	1550	tags=17%,	3117/917/3458/4794/10148/30009/915/4792
0.001013993	0.006057996	2123	tags=20%,	245972/1960/718/1026/1959/5366/4792/718
0.00811359	0.025621864	2540	tags=27%,	2266/2244/2243/91807/10125/6916/7408/22
0.004060914	0.016321376	3271	tags=28%,	3576/6347/925/7124/5880/7409/4792/7186/
0.002227171	0.010702655	1541	tags=16%,	3117/6401/3383/925/3684/3683/7412/3689/
0.025963489	0.063769973	2786	tags=24%,	948/10319/6696/5649/7059/3371/1311/3679
0.047171738	0.105327644	2813	tags=30%,	399694/391/1759/4233/8936/9564/5296/367
0.002631579	0.012007907	3225	tags=26%,	3576/1960/1026/1959/7124/6775/4792/7097
0.006072874	0.020652691	2663	tags=22%,	245972/54/23659/51172/1522/7805/4125/23
0.021010101	0.053383197	2071	tags=24%,	5054/1026/7076/3458/5209/3162/1906/6198
0.003442689	0.014393101	1474	tags=16%,	9021/1026/5292/3587/3458/6775/3600/3575
0.02494423	0.062658952	4043	tags=30%,	245972/5464/23545/9114/27068/245973/135
0.04640749	0.10467855	3709	tags=31%,	1026/330/10319/1164/4792/4616/7186/4609
0.011140369	0.031571895	2786	tags=22%,	330/10319/399694/91807/5880/6696/7409/5
0.0257222	0.063769973	1887	tags=21%,	5091/2806/275/1737/84693/3417/26275/647
0.028638682	0.068811478	2922	tags=24%,	64241/570/1576/6548/477/10998/10864/524
0.019133151	0.04975804	2634	tags=24%,	6548/477/7137/7169/7381/489/4624/784/65
0.036153996	0.084125643	1852	tags=23%,	3945/92483/1737/217/128/669/2026/130/22
0.009929907	0.030122072	1722	tags=37%,	10455/30/8540/1384/6342/10654/51703/929
0.010735897	0.030820757	4864	tags=48%,	80854/55904/2145/2639/223/11105/83852/6
0.020360675	0.05233466	1697	tags=28%,	873/6817/1577/4259/2948/1558/2949/37315
0.014986376	0.04039973	1586	tags=33%,	4129/217/64577/55526/11185/847/224/1892
0.002331908	0.010967542	2014	tags=26%,	383/162417/5091/2806/27430/435/3417/647
0.004502741	0.017773977	1136	tags=25%,	1577/128/4259/2948/2949/130/27294/37315
0.005429513	0.019358197	2214	tags=33%,	1158/383/2806/4129/58510/217/224/8974/2
0.00155129	0.008119472	1822	tags=33%,	1629/11112/84693/217/26275/5095/549/569
0.041868579	0.095414017	1546	tags=35%,	8876/217/60496/5169/224/53354/1807
0.005974176	0.02063449	1852	tags=37%,	3945/1629/92483/84693/26275/5095/1892/1
0.004817884	0.018468949	3600	tags=47%,	51733/8310/2571/57571/8639/217/26275/22
0.03962704	0.091246473	1737	tags=41%,	275/10840/7298/6470/160428/4522/10841
0.007354364	0.023907376	2916	tags=37%,	38/2752/54363/275/84693/6470/5095/847/5
0.012521672	0.034172205	2615	tags=36%,	2744/477/762/27165/5105/8671/5106/482
0.012521672	0.034172205	2916	tags=41%,	38/2571/79944/7915/6296/1892/18/54988/1
0.000579374	0.003916364	3011	tags=46%,	57494/2571/2752/2744/80150/2806/435/791
0.000195236	0.001754804	1136	tags=33%,	1577/128/4259/2948/2330/1558/2949/130/2
0.015928516	0.042422173	1731	tags=47%,	9104/9365/2990/217/224/55586/79799/5457
0.005393951	0.019358197	2167	tags=45%,	57571/3176/4129/217/224/10841/26/144193
0.000193125	0.001754804	1737	tags=43%,	5723/23464/8639/275/4129/6470/669/26227
0.0048459	0.018468949	2800	tags=50%,	2752/2744/383/162417/2806/435/445/27165
0.005093046	0.019081885	2901	tags=71%,	2571/2328/570/124975/2686/1036/2330/232

7412/3689/6403/7097/7059/1311/4233/3569/3592/3606/2994/3586/2532/958/975
83/6347/6352/2921/7124/3458/3683/3600/54/4312/4050/3689/942/7097/23545/9114/3119
3306/929/4792/7097/3569/4791/4790/834/3592/3606/3303/836
59/3689/929/714/4843/3569/4790/715/3654/148022/3678/834/3592/1072/716/23643/727/836/3586
921/7124/7132/7412/4050/929/4792/4616/695/7186/55367/5743/6850/4067/4791/4790/3654/4055/1
/9021/6352/330/1051/2921/1326/7124/602/7132/6376/3600/3726/7412/3659/1906/4792/7186/5606/
92/1445/23545/9114/6868/4067/4233/4790/245973/10312/529/9296
9/3683/629/3689/6403/3880/728/3119/714/2357/715/3872/2358
3586/941/958

/9296/6521/10723/528/127124/534/535
/715/6628/733/716/727/3586/941/958/717
69/3592/3606/5579/3586/8542/356/4615/5332
374/3932/23495/958/926/7535
/1051/2921/7124/2354/3458/4312/4792/7186/27190/5743/3569/4790

/1524/6352/2921/1230/7124/3587/7132/6376/8807/8797/2826/3561/8764/1436/6369/3569/6355/405
5798/1363/941
4/3458/7132/915/4792/7097/714/7046/2769/4843/3569/4790/3654/5296/148022
43/3684/1675/3687/719/629/3689/728/5055/3080/714/2157/7035/715/5329/733/3053/716/5328/727
8/912/3689/929/911/7097/910/2769/4843/3569/338382/4790/3911/5296/5272/733/5568/3592/5579/
4792/942/7097/5606/51284/7096/3455/5605/3569/4790/3654/5296/148022/3627/3592/29110
551/1981/1756/836/60/941/857/958/3908
7/5715/5690/5704/5701/10197/5694/5686/143471/5684/5682/23198/5692/5695

536/1356/440738/6303/64116/84557/2730/2512/2879/5093
10803/6370/3586/941/23495/958
/3119/1520
43/7124/1675/719/7132/200916/1839/629/4312/4792/6403/7097/728/714/6868/51284/6850/3455/35
/914/915/911/910/3119/3563/1436/3569/3574/3566/3678
366/10105/6403/7097/728/51284/6850/5605/2357/4790/2358/5296/1535/834/5579/1536/727/820/60
/84790/11151/8685/6441/7277/5289/7097/7059/6890/1311/23545/9114/3119/1520/338382/715/2459
448/666/331
/1524/6352/2921/1230/6376/399694/5880/7409/4792/3055/2826/23533/56288/6369/4067/2783/109/

9296/5568/11015/60/533/528/127124/534/535/30001/9601/29927/6558/50617/527/23480/375

22/5434/834/5441/29110/3606/103/81030
3119/5743/4843/4790/3654/1535/3592/5579/1536
1230/7132/100507436/4792/3055/942/5289/7186/23533/637/4609/5743/6850/4067/2783/3455/5605/
/4792/10105/7097/5606/23533/3119/4843/4790/3654/3911/79444/3949/3592/23643/3918/3303/572/
02/100507436/3689/962/2207/3823/8797/637/6850/3455/5605/5551/80329/4773/5296/7305/5579/39
/7132/4792/8797/5646/3119/637/90865/10898/51284/3455/56000/5605/3569/4790/64135/5296/1480
/5292/7412/1906/6776/1729/7046/3569/4790/5590/5296/50507/5579/113026/1536/836/3265
9/7132/929/4792/5606/728/23417/714/3569/4790/3654/50507/148022/3592/29110/23643/727/836
8/79659/9230/51164/5878/8655/140735/10488/9586
3726/2274/2355/4792/695/7186/54209/4982/1436/6850/7046/3455/4791/4790/4773/11006/5296/153
9/3561/3569/4790/50615/3566/3592/3606
92/7097/5606/6198/5605/29126/4790/4773/5296/148022/238/3932/6199/3265/10538/4215/4893/479
50/4067/5605/4790/4773/11006/5296/3635/5579/3265/4893/4793/11027/975/933/5534/8503/11024
7/3458/7132/3689/3656/929/8877/11151/2207/4046/5289/7097/23545/9114/3119/637/1520/6850/70
9/2180/7186

436/4609/5605/4790/5296/5914/6199/572/3265
4790/4616/7277/8797/7186/637/4170/3563/55367/1520/5605/5551/1676/1439/823/4790/3710/84823
794/11047/915/4792/4616/695/7186/7097/8900/6890/5606/3119/637/4609/6850/4067/3455/3569/47
842/1848/929/79058/4616/64332/942/4291/8900/3087/1436/3486/3398/4609/4804/84444/4233/3569
915/4792/5605/4790/4773/5296/5063/3932/3265/3586/4893/4793/926/5603/5534/8503/7535
792/3119/3561/6776/4790/4773/3566/3592/4851/3932/3714
7124/115362/4792/10135/7186/3455/3569/4790/3710/51393/148022/1535/7205/834/7188/29110/360
5743/6850/3569/4791/4790/3710/4773/5296/834/3592/3265/6367/3586/4893/29108/22808/5603/553
/3119/3561/6776/7046/3569/4790/4773/50615/3566/5914
6/8900/2968/9114/2965/6776/55697/6850/4067/991/4791/5423/4790/4055/5296/8850/5568/84699/7
07/695/23533/83706/5499/6850/4067/84876/109/3710/5590/5296/108/54518/112/5568/8673/6786/8
5606/391/5605/3569/4790/3654/9564/4773/5296/148022/3678/834/29110/3606/3932/81873/60/9138
914/3385/1364/6403/942/3119/9076/57502/64101/29126/8506/1462/3680/23114/7122
/1292/3694/3911/3680/3678/1298/3918/3690/7450/3908/3693/2335
8/8218/859/81873/60/3059/10093/857/1213/79767/6464/2335/1785
/8900/5606/637/6776/4609/7046/5605/3569/4790/3654/4773/64135/5296/1871/148022/84699/29110
545/9114/1520/8692/10053/9516/27074/1174/10312/2588/8218/10717/162/8906/4669/8722/9179/30
/3948/4843/5605/3569/4790/8569/3101/4055/5296/3098/2023/5579/5214/9470/1536/2872/6199
/9655/4170/3561/1489/6776/3563/8027/4609/3455/3569/1439/3601/50615/5296/3574/3566/1443
2/10312/1349/529/9296/9377/4707/4706/126328/533/4701/4708/528/51079/127124/534/4714/535/4
/5743/4843/4790/3911/5296/1871/79444/7188/3918/836/1643/3908/2335/317/8503/1030/331/7185/
649/7408/2316/54776/7059/3371/1311/5499/1729/3679/2317/1292/4233/3694/9564/3911/3680/5296
0/5095/128/5634/847/2026/84706/26227/1892/4329/55753/29968/2203/229/51179
3/1244/343/8671/6554/482/200931/79799/54578/9376/6555
46/785/1345/84701/776/7171/7139/4634/9254/55799/482
4/5105/5106/130589/2203/229
60/8310/84188/54677/570/54363/10478/3295/55711/3417/8800/5827/847/55825/2053/373156/1962/
4324/79813/55870/58508/51111/51166/4297/38/6419/56950/217/79723/55526/2122/64754/8424/224
6/2947/79799/54578/1551/2938
/7166/26/1962/8564/130013/3620
0/445/5634/2026/84706/26227/384/29968/229/5053
6/22977/2947/79799/54578/2938
6/384/84735/1610/2628/79814/112817
22/36/224/1892/18/4329/1962/64902

8/4329/1962/79611
4/1892/18/4329/1962/84735/1807

1179/112817

962
5/445/27165/84706/18/56954/443/2346/64902
329/373156/2947/79799/54578/2938/2326/2327
8
/84735/443
/635/1610/2628/29958/29968/51268/64902
/84706/384
9/2326/2327

48022/7188/5579/3554/23643/5328/3932
5743/3569/4790/5296/3627/84699/7188

5/5197/3627/10803/3606

/7450/717/5327/623/5627/710/624
3554/3918/836

69/4790/715/3654/6227/64135/5296/3627/733/834/3592/29110/5579/1536/716/727/103

/3690/7450/10014/291/5603/9759/8503/5332
73/10312/1535/529/3678/9296

4790/9564/6355/5590/5296/5197/108/112/3627/5568/10803/2787/5579/2792/572/3265/3577/6367/6

3569/4790/3710/4773/5296/1871/148022/2787/29110/2792/836/3265/440738
836/3586/958/4793/3312/3908/3717
32/836/3265/4893/6464/79465/369/5534/10870/356/8503/7535/3459
22/3627/834/3592/29110/3606/5579/103/836/1965/60/8480

5/7305/3554/3932

3/3717/5603/5534/4615/8503/7535/3459

96/4843/3569/4790/3654/10312/8625/3592/3606/572/820/836/8767/1594/3586

/9451/5296/10039/5783/572/836/4000/3265/1965/60/8722
91/4790/5718/3654/5714/5296/953/1871/3627/6892/7188/29110
/4790/5914/604/2120/5328/51513/1643/5090/1669/6495/958/3728/8148/5327

6/1536/81858/820/8767/440738/10010/5585/1669/4793/2634/29108/25828/84557/5603/5027/4615/5[illegible]
4

188/2648/85477/572/836/3265
5366/60/9138/3690/7450/10672
/3586/5585/10093/2335/29108/926/5603/8395/4615/8503/7535

/5579/572/836/3265/1643/4893/2002/1642/3717/317/369/5603/356/4615/8503
73/23163/3988/1213/533

697/64077/4695/4696/7384
7187
/5063/4636/3678/1298/5579/859/85366/3918/572/3265/60/3690/857/7450/2002/6464/3908/3693/23[illegible]

5190/1610/51268/51179/11001
/123688/1892/6839/1962/51268

[illegible]370/2931/4893/1794/4793/10681/6464/409/6357/3717

[illegible]332/64127

[illegible]35

IL1B

ID	Descriptic	setSize	enrichment	NES	pvalue
hsa05323	hsa05323	Rheumatoic	84	0.69779945	2.557434698
hsa05144	hsa05144	Malaria	46	0.694891454	2.279405943
hsa00260	hsa00260	Glycine, s	35	0.681503534	2.112481674
hsa00360	hsa00360	Phenylalar	16	0.680160422	1.758607589
hsa05143	hsa05143	African tr	34	0.664229131	2.049181296
hsa04657	hsa04657	IL-17 sigr	90	0.653977758	2.430299997
hsa04668	hsa04668	TNF signal	109	0.650638761	2.492902068
hsa05310	hsa05310	Asthma	26	0.647026669	1.881060061
hsa00270	hsa00270	Cysteine a	46	0.642156753	2.106423832
hsa05332	hsa05332	Graft-vers	34	0.63928256	1.972219829
hsa00650	hsa00650	Butanoate	22	0.631821568	1.770667092
hsa05134	hsa05134	Legionellc	53	0.611781042	2.065940649
hsa01523	hsa01523	Antifolate	28	0.610783729	1.809011146
hsa04064	hsa04064	NF-kappa E	98	0.608076052	2.293802397
hsa04061	hsa04061	Viral prot	91	0.606685405	2.257378143
hsa04940	hsa04940	Type I dia	38	0.60257098	1.900258318
hsa00100	hsa00100	Steroid bi	19	0.600858381	1.621596777
hsa00020	hsa00020	Citrate cy	28	0.597138075	1.76859563
hsa00380	hsa00380	Tryptophar	42	0.589998182	1.897224625
hsa04620	hsa04620	Toll-like	93	0.582842077	2.176086332
hsa04614	hsa04614	Renin-angi	22	0.58061513	1.627162092
hsa05219	hsa05219	Bladder ca	41	0.572014573	1.832087317
hsa05120	hsa05120	Epithelial	69	0.565666822	2.012499727
hsa00770	hsa00770	Pantothena	20	0.563727679	1.54027096
hsa05321	hsa05321	Inflammatc	61	0.56048864	1.945058012
hsa05330	hsa05330	Allograft	33	0.546860416	1.671781324
hsa05164	hsa05164	Influenza	151	0.530737569	2.135727516
hsa00051	hsa00051	Fructose a	30	0.5295189	1.587680523
hsa00601	hsa00601	Glycosphir	27	0.525052609	1.543947174
hsa00030	hsa00030	Pentose ph	29	0.522016906	1.555838952
hsa04612	hsa04612	Antigen pr	63	0.521015022	1.81776871
hsa00052	hsa00052	Galactose	30	0.516503606	1.548656177
hsa05416	hsa05416	Viral myoc	55	0.515678037	1.752118197
hsa04933	hsa04933	AGE-RAGE s	99	0.503266659	1.902089672
hsa05418	hsa05418	Fluid shea	128	0.500171516	1.963509871
hsa04623	hsa04623	Cytosolic	54	0.497022572	1.683222867
hsa04960	hsa04960	Aldosteror	36	0.49432154	1.539929173
hsa05140	hsa05140	Leishmania	66	0.491220608	1.732998451
hsa04622	hsa04622	RIG-I-like	64	0.486874283	1.705771738
hsa00250	hsa00250	Alanine, a	35	0.476449964	1.476869551
hsa04625	hsa04625	C-type lec	102	0.473883365	1.796702711
hsa04672	hsa04672	Intestinal	41	0.472799182	1.514313491
hsa04640	hsa04640	Hematopoie	89	0.470803223	1.745142331
hsa00350	hsa00350	Tyrosine m	36	0.469976745	1.464089347
hsa04062	hsa04062	Chemokine	179	0.464457303	1.905906064
hsa00071	hsa00071	Fatty acic	41	0.46199103	1.479696405
hsa05320	hsa05320	Autoimmune	44	0.461963911	1.501740453
hsa00480	hsa00480	Glutathior	46	0.460570767	1.510779471
hsa05142	hsa05142	Chagas dis	98	0.460276095	1.736267044
hsa00010	hsa00010	Glycolysis	62	0.456542668	1.589285225
hsa03320	hsa03320	PPAR signa	69	0.45481865	1.618129921
hsa05150	hsa05150	Staphylocc	72	0.450176615	1.608496831

hsa05167	hsa05167	Kaposi sar	183	0.450129654	1.852672009
hsa00330	hsa00330	Arginine a	46	0.449362916	1.474015109
hsa04662	hsa04662	B cell rec	78	0.447886781	1.623523824
hsa05146	hsa05146	Amoebiasis	100	0.445092077	1.68433684
hsa04146	hsa04146	Peroxisome	79	0.444781047	1.611686462
hsa05161	hsa05161	Hepatitis	154	0.444164216	1.791745576
hsa04664	hsa04664	Fc epsilon	63	0.439395786	1.533007449
hsa04613	hsa04613	Neutrophil	102	0.434426287	1.647103369
hsa04621	hsa04621	NOD-like r	164	0.434392453	1.762192918
hsa04924	hsa04924	Renin secr	67	0.433772801	1.531841247
hsa04659	hsa04659	Th17 cell	103	0.428134031	1.625325103
hsa04380	hsa04380	Osteoclast	120	0.422005485	1.639319273
hsa01200	hsa01200	Carbon met	107	0.421062559	1.608736703
hsa05169	hsa05169	Epstein-Ba	187	0.417750086	1.725925808
hsa04514	hsa04514	Cell adhes	143	0.413789012	1.649406859
hsa04650	hsa04650	Natural ki	112	0.410788318	1.578493418
hsa05171	hsa05171	Coronaviru	171	0.408856363	1.668514166
hsa04936	hsa04936	Alcoholic	133	0.405439395	1.599879709
hsa05230	hsa05230	Central ca	67	0.40399644	1.426687909
hsa04658	hsa04658	Th1 and Th	87	0.403012572	1.487688218
hsa05152	hsa05152	Tuberculos	166	0.396347263	1.612212632
hsa04210	hsa04210	Apoptosis	129	0.392383913	1.541155848
hsa05135	hsa05135	Yersinia i	130	0.391326217	1.540663878
hsa05202	hsa05202	Transcript	164	0.38774975	1.572978211
hsa01240	hsa01240	Biosynthes	132	0.386601444	1.524782909
hsa04670	hsa04670	Leukocyte	105	0.376985251	1.435504845
hsa05145	hsa05145	Toxoplasmo	106	0.374424891	1.427219494
hsa04932	hsa04932	Non-alcoho	143	0.349799832	1.394339206
hsa04068	hsa04068	FoxO signa	127	0.342250005	1.341619605
hsa05170	hsa05170	Human immu	197	0.330484283	1.372020393
hsa05207	hsa05207	Chemical c	175	0.323852419	1.324952717
hsa05130	hsa05130	Pathogenic	182	0.314755046	1.293309657
hsa04512	hsa04512	ECM-recept	85	-0.368872948	-1.425656346
hsa00450	hsa00450	Selenocomp	15	-0.623139964	-1.623583619
hsa00430	hsa00430	Taurine an	14	-0.62696815	-1.609795811

p.adjust	qvalues	rank	leading_ec	core_enrichment	
0.000177179	0.000177179	0.002459162	1503	tags=36%,	3576/2920/3553/6347/6364/2459
0.000187091	0.000187091	0.002459162	1270	tags=35%,	3576/6401/3553/6347/3383/7124/
0.000188573	0.000188573	0.002459162	879	tags=29%,	29958/2628/29968/51268/1610/6
0.006440281	0.006440281	0.02914347	2061	tags=38%,	3242/5053/1644/259307/2805/21
0.000188111	0.000188111	0.002459162	1415	tags=29%,	6401/3553/3620/3383/7124/3827/
0.000176149	0.000176149	0.002459162	1715	tags=28%,	3576/2920/3553/6347/6364/3627/
0.000173822	0.000173822	0.002459162	1821	tags=33%,	6401/2920/3553/6347/6364/3627/
0.002499039	0.002499039	0.016294902	2150	tags=50%,	3115/7124/3117/3119/958/3123/
0.000187091	0.000187091	0.002459162	2256	tags=37%,	29968/23743/635/64902/259307/
0.000188111	0.000188111	0.002459162	3817	tags=74%,	3553/3115/942/7124/3117/3119/
0.007337324	0.007337324	0.030932488	1928	tags=27%,	54988/6296/1962/3033/38/3157
0.000184706	0.000184706	0.002459162	1891	tags=26%,	3576/2920/3553/7124/2921/836/
0.003803728	0.003803728	0.021258929	1392	tags=32%,	3553/1147/8836/7124/6470/239/
0.000174734	0.000174734	0.002459162	504	tags=15%,	3576/6351/2920/3553/597/5336/
0.000176274	0.000176274	0.002459162	1188	tags=25%,	3576/6351/2920/6347/6364/6373/
0.001131435	0.001131435	0.01021498	2623	tags=45%,	3553/3115/942/7124/3117/3119/
0.024485037	0.024485037	0.085892144	2253	tags=42%,	1591/1594/6307/7108/10682/171
0.006085964	0.006085964	0.02914347	2418	tags=36%,	55753/5106/8801/3418/5105/174
0.000754148	0.000754148	0.007376095	1873	tags=40%,	64577/3620/130013/8942/1543/1
0.000176429	0.000176429	0.002459162	1137	tags=19%,	3576/6351/3553/6373/3627/1147/
0.023749759	0.023749759	0.085892144	976	tags=18%,	5972/59272/290/183
0.001689506	0.001689506	0.013338836	1334	tags=22%,	3576/4312/4609/4318/999/7057/
0.000179986	0.000179986	0.002459162	1503	tags=20%,	3576/2920/5336/245972/1147/40
0.039596273	0.039596273	0.115048782	2447	tags=45%,	1807/1806/224/53354/586/51733/
0.000364232	0.000364232	0.004071369	2240	tags=34%,	3553/6775/3115/7124/3117/3119/
0.011972634	0.011972634	0.048455488	3745	tags=58%,	3115/942/7124/3117/3119/958/3
0.000168294	0.000168294	0.002459162	2150	tags=24%,	3576/3553/6347/3627/5340/1147/
0.024672613	0.024672613	0.085892144	3054	tags=40%,	2203/229/57016/3101/3795/3099/
0.034324943	0.034324943	0.1038324	2142	tags=26%,	84002/2651/6489/10678/2523/10
0.032313248	0.032313248	0.099804078	2909	tags=31%,	2203/229/2539/414328/9104/521
0.001277372	0.001277372	0.01110542	4349	tags=54%,	10437/3115/7124/3117/3119/380
0.031694819	0.031694819	0.099804078	2510	tags=40%,	8972/130589/57016/2717/3101/3
0.002572111	0.002572111	0.016318087	3843	tags=49%,	3115/942/3383/3117/637/3683/3
0.000174338	0.000174338	0.002459162	1916	tags=25%,	3576/6401/3553/6347/5336/3383/
0.000171174	0.000171174	0.002459162	2755	tags=27%,	6401/3553/6347/2938/1147/3383/
0.004784689	0.004784689	0.025020804	147	tags=7%,	6351/3553/3627/1147
0.028495943	0.028495943	0.092903439	2720	tags=28%,	3758/3291/6340/3479/481/9351/
0.001986635	0.001986635	0.014131409	2878	tags=32%,	3553/3115/7124/3117/3119/1915/
0.003271538	0.003271538	0.01919876	182	tags=6%,	3576/3627/1147/7124
0.043183104	0.043183104	0.120674589	4006	tags=49%,	64902/445/259307/2747/84706/7
0.000524751	0.000524751	0.00535558	1392	tags=15%,	3553/1959/5336/1960/1147/7124/
0.030035667	0.030035667	0.096581886	1479	tags=29%,	3115/942/3117/3119/958/3600/3
0.002115283	0.002115283	0.014603966	3009	tags=36%,	3553/3115/290/7124/3117/3563/
0.045480279	0.045480279	0.12559879	2231	tags=28%,	3242/7306/1644/259307/130/280
0.000168719	0.000168719	0.002459162	1503	tags=16%,	3576/6351/2920/6347/5336/6364/
0.039797259	0.039797259	0.115048782	2525	tags=34%,	23205/1962/30/3033/130/224/21
0.034502156	0.034502156	0.1038324	2932	tags=41%,	3115/942/3117/3119/958/3123/3
0.031992516	0.031992516	0.099804078	1051	tags=20%,	2938/9027/2539/290/4257/3418/
0.001572602	0.001572602	0.013183842	1801	tags=19%,	3576/3553/6347/1147/7124/3827/
0.00874795	0.00874795	0.036025721	2231	tags=29%,	2203/229/130589/5106/92483/51
0.00737941	0.00737941	0.030932488	1056	tags=17%,	11001/1582/2170/4312/23205/21
0.007181329	0.007181329	0.030932488	1491	tags=25%,	1672/728/5340/3115/3383/3117/

0.000168407	0.000168407	0.002459162	2752	tags=23%,	3576/2920/5336/1147/4609/942/
0.0404116	0.0404116	0.115048782	2396	tags=28%,	2628/1610/79814/262/58510/224/
0.004796589	0.004796589	0.025020804	2723	tags=28%,	5336/1147/4067/10288/10859/10
0.001921062	0.001921062	0.014092	1916	tags=22%,	3576/2920/3553/7124/5272/910/
0.005177647	0.005177647	0.025563779	1670	tags=19%,	6648/51268/1610/11001/3418/83
0.000338295	0.000338295	0.003970515	2900	tags=23%,	3576/1959/1960/100506742/6775/
0.016970803	0.016970803	0.066394545	1857	tags=24%,	5336/7124/4067/10451/8605/588
0.003498338	0.003498338	0.020028997	2625	tags=25%,	5336/728/366/3683/2357/6403/1
0.000508388	0.000508388	0.00535558	2766	tags=21%,	3576/2920/3553/6347/2635/1005
0.015187127	0.015187127	0.06042336	1899	tags=18%,	5972/116/3759/1906/154/183/91
0.004551024	0.004551024	0.024844023	2662	tags=24%,	3553/1147/3115/3117/3119/4794/
0.002252643	0.002252643	0.015107948	3230	tags=28%,	3553/5336/1147/7124/8061/7305/
0.005227392	0.005227392	0.025563779	3573	tags=35%,	29968/2203/229/2539/2731/5575
0.000334784	0.000334784	0.003970515	2900	tags=27%,	5336/3627/1147/4609/3115/3383/
0.001704739	0.001704739	0.013338836	2878	tags=31%,	6401/3115/942/3383/3117/3683/
0.005213764	0.005213764	0.025563779	4099	tags=38%,	5336/962/3383/7124/637/3683/7
0.001011463	0.001011463	0.009497108	1137	tags=12%,	3576/3553/6347/5336/728/3627/
0.002729444	0.002729444	0.016428232	2517	tags=22%,	3576/2920/3553/728/1147/2168/
0.040679805	0.040679805	0.115048782	2740	tags=31%,	4609/2539/6514/92483/3418/999
0.018364824	0.018364824	0.070670506	3158	tags=28%,	6775/1147/3115/3117/3119/864/
0.001856853	0.001856853	0.014060385	2755	tags=25%,	3553/245972/3115/7124/3117/35
0.006322625	0.006322625	0.02914347	2336	tags=23%,	597/1515/100506742/1147/7124/
0.006455997	0.006455997	0.02914347	1891	tags=15%,	3576/3553/6347/1147/7124/6195/
0.002711405	0.002711405	0.016428232	1644	tags=17%,	3576/597/4609/942/3398/9915/6
0.007157464	0.007157464	0.030932488	2505	tags=26%,	3242/29968/3620/8836/1503/894
0.024522683	0.024522683	0.085892144	1521	tags=18%,	5336/3383/3683/4318/58494/907
0.025703794	0.025703794	0.087443877	1680	tags=22%,	1147/3115/7124/3117/3587/330/
0.022332083	0.022332083	0.083208932	2052	tags=12%,	3576/3553/7124/637/1050/836/4
0.047194096	0.047194096	0.127335554	4013	tags=31%,	6648/1147/1901/8743/10365/510
0.021308725	0.021308725	0.080676496	2852	tags=21%,	5336/1147/7124/637/200315/200
0.037512673	0.037512673	0.111463373	3340	tags=21%,	2938/9314/407026/4609/1555/15
0.047023004	0.047023004	0.127335554	2662	tags=18%,	3576/3553/1147/7124/9071/8743/
0.026406429	0.026406429	0.088550884	1010	tags=14%,	22801/7058/3690/1278/3908/333
0.02488176	0.02488176	0.085892144	239	tags=20%,	11185/9060/114112
0.026966292	0.026966292	0.08915468	840	tags=29%,	51380/2687/2328/2327

72/4312/3115/942/3383/7124/3117/3683/3119/54/2921/50617/3600/4314/6372/3123/3111/3109/709
/3683/6403/958/7412/7057/7097/3569/7042/6382/4233
35/64902/2731/6470/55349
3
/7412/3569/169355/355
/4312/1147/7124/4318/8061/7128/2921/6354/836/55540/4314/6372/5743/3569/5970/2919/6279/559
/1147/3383/7124/3659/4318/3726/330/7128/2921/7412/6376/1906/836/3600/4314/6372/7424/5743/
3111/3109/3113/2207/3122/2205/3108
92483/2729/262/56267/144811/2805/84245/23382/586/3948/4507/2730
3802/3123/3824/3111/3109/3569/3113/355/3122/3552/3108/3133/3106/5551/3134/3135/3812/941/3

1915/7097/3569/5970/2919/664/3303/834
3714/3569/5970
1147/3383/7124/4067/330/7128/2921/958/7412
/3627/6355/7124/9547/3587/8743/1524/2921/6376/4283/1234/6354/1230/57007/6372/3569/7133
3123/3111/3109/3113/355/3122/3552/3108/3133/3382/3106
3/6713/6646
3/6392/8802/48/3417
344/6999/8564/259307/1962/56267/3033/224/1743/169355/38/219
/942/6696/7124/7098/958/4283/51311/7097/5606/5605/5604/3569

5605/5604/4893
57/58494/2921/50617/836/4233/525/5970/2919
/219/79717/80347
/3123/3111/3109/7097/3569/3113/7042/64127/50615/5970/3122/3561/3552/3108/149233
123/3111/3109/3113/355/3122/3108/3133/3106/5551/3134/3135/941
/3115/3383/7124/3117/637/7098/56000/3119/8743/3838/836/3337/3123/3111/3109/5605/5604/3569
/5209/5211/5214/226/5210/5207
317/8707
1/5214/226/8277
2/3123/3824/3111/3109/1520/3113/1508/3122/3303/6890/3108/6892/3133/567/3106/5993/3823/331
906/3099/6476/5211/2720/5214/2584
119/958/836/3123/5880/3111/3109/3113/3122/1604/3108/3133/3106/3689/5551/595/3134/3135/588
/7124/7412/7056/1906/1958/836/7424/183/3569/7042/4893/5970/50507/4846/1729/23236/3552/850
/7124/445/4318/10365/4257/7412/7056/1906/6385/5880/5155/2949/91860/8878/6382/5970/4846/85

3503/482/3845/483
/3123/5743/3111/3109/7097/3113/7042/5970/3122/3552/3108/4688/3725/3689/3676

90/2805/2744/435/158/27165/9945/2875/5471/8659/2746/18
/3659/4046/1263/5743/91860/3569/2207/4893/5970
123/3111/3109/3569/3113/3122
3119/910/960/913/3123/3111/3109/917/3569/3113/3122/100133941/915/1440/3552/1604/3108/933/
5/220074/1638/218/124
/6373/3627/6355/1147/9547/4067/1524/3055/2921/6376/10451/4283/1234/6354/1230/6372/5880/56
30/38/219/34/124/51703/2181/36
111/3109/3113/355/3122/1081/3108/3449/3133/3106/3442/5551
2729/2878/2949
/9630/2769/7097/917/3569/7042/5970/355/5519/8837/915/23236/8503
05/130/3101/224/3948/3099/219/2023/10327/5211/218/5214/124
58/5106/5105/8309/1962/30/2710
3683/2357/6403/3119/629/1675/3123/3111/3109/3113/3122/719

3383/4067/637/7098/3055/2921/1234/1230/836/7538/2247/5743/5155/5605/5604/91860/3569/4893/
/2805/4846/112483/219/6303/5033/112817
451/11024/4794/118788/5880/5605/5604/4893/5970/7409/8503/11027/933/5534/3845/3725/10892
2921/9630/913/836/7879/2769/7097/3569/7042/5970/2919/338382/23236/8503/5566/1288
09/1962/5194/26061/30/5189/51179/10005/2180/5827
/1147/4609/7124/637/7098/4318/836/7097/5606/5605/5604/3569/7042/4893/8900/5970/355/7534/2
0/5606/5605/5604/2207/4893/3937/7409/8503/2205
184/10105/51311/5880/7097/5605/5604/2243/5970/1183/23236/8503/834/51284/2266/4688/820/839
06742/2633/1147/7124/330/7128/2921/115361/2634/115362/3569/64127/5970/1508/2919/10010/232
360/5136/1508/5732/23236/5566
/10148/3123/3111/3109/917/3569/3113/50615/9466/5970/3122/915/3561/3108/149233/5602/5534/3
/3726/10288/54/4286/10859/11024/10326/5604/8878/7042/5970/3937/3552/8503/11027/7006/4688/
3/6470/8801/3418/2747/4329/84706/414328/3101/51179/1743/2805/3099/38/6392/2023/9104/5211/
/7124/3117/4067/637/3683/3119/7128/864/958/3280/960/4794/836/3123/3111/3109/7097/5606/917
6403/58494/3119/6402/9071/1462/9075/958/7412/9076/999/6385/9073/5788/3123/3111/3109/3113/
305/8743/117157/10451/836/3802/3824/5880/5605/5604/2207/4893/3937/355/7409/8503/3449/8032
6192/59272/4312/1147/7124/7098/6403/629/51311/1675/140801/4314/7097/3569
7124/10891/2921/836/130/5606/3569/224/5970/355/719/50507/2919/219/34/1499/8772/124/23411/
7/3101/5605/5604/4233/4893/3948/3099/8503/5211/2744/5214/3417/3845/9123/6510
4794/3123/3111/3109/917/3113/5970/3122/915/3561/3714/3108/5602/5534/3725/3566/84441/5588
37/637/3687/3119/1594/4046/1263/3656/50617/836/7879/3123/3111/3109/7097/1520/91860/3569/3
637/330/3563/1512/8743/1075/10038/1439/836/1520/5605/5604/4893/7846/5970/355/1508/8837/79
/10451/5880/5606/5605/5604/3569/8395/382/5970/3937/7409/6197/8503/834
4332/4318/330/3248/1050/4286/958/8842/3087/3479/4066/4314/2530/3569/894/6756/4233/7102/89
2/6999/6470/145226/8564/2729/11019/2643/112724/790/79799/224/169355/53354/4830/129607/549
1/29119/9075/7412/9076/10451/103910/9073/7408/5880/11069/7122/7409/1364
3119/958/3949/1234/10105/836/3123/3111/3109/7097/5606/3113/7042/345611/5970/3122/3303
725/3569/4698/5970/355/4716/1347/3552/8503/6392/1537
6/1263/3479/5105/1032/5605/5604/3569/894/7042/4893/664/2033/10110/8503/23411/5602/2308/85
316/1234/836/5880/7097/5606/917/5605/5604/91860/8905/7133/4893/60489/5970/355/915/10053/8
43/4257/6195/3838/154/2247/54852/2949/79799/5605/5604/4893/5970/6197/8503/148327/5566/114
/9075/9076/9871/9073/836/4542/3569/3071/382/7122/7846/26999/5970/355/1364/79861/834/8772/
9/3910/8515/1311/2335/1293/1277

7/3569/3113/7042/525/3122/2919

7/1440
5606/5604/3569/7133/64127/5970/355/8837/2919/3976/8503/148327

804

/3113/5970/355/3122/2033/3552/8503/834/56649/51284/8772/3108

0/3134/3135/3812/5641/3804/3458/8625/3320/6891/1514

1/941/4625
3/1288
8/3552/8503/1499/4780/4688/5602/3685/657/3554/3725/596

3554/3678/2208/1435/3676/945/914/3566

04/4893/5970/7409/2919

5970/355/2919/2033/8503/1499/8772/64422/2791/5294/5602/3449/5534/3133/3845/2793/3106/3725

033/8503/148327/8772/5602/3449/3845/3725/3442/596/90993/64135/1871

33/9734
36/834/11035/8772/5602/3449/9474/820/1669/3725/59082/3442/596/23710

554/3725
5602/5534/3554/4982/3725/9846/1435/55423/126014/54209/2885
5214/8802/48/3417/283871/26275/226/8277/4199/5230/8310/2875/9563/1738
/3569/3113/894/8900/5970/355/3122/915/8503/6890/8772/3108/5602/3449/6892/5709/3133/567/31
152404/94030/6382/7122/3122/1364/214/22865/4359/139065/3108/4684/3685/933/3133/3106/8506/
8/5534/3133/3845/3106/3442/3689/5551/2885/3823/6452/3135/3812/5881/3804/4772/4277/8795/34

5602/3449/2308/10645

113/2207/7042/64127/5970/3122/22925/2033/3552/8772/3108/5602/3449/820/5534/5993/10892/344
861/8503/4170/1522/8772/5602/7185

00/5970/2521/4005
95/7390/586/219/205/9104/10327/57026/2730/7358/79717/80347/124454

417/3845/1950/23710/26260/595/2885/6446/9133/5563/4088/11345/5565/5562/1454/4089/7046
503/6890/8772/2791/5602/3449/6892/7465/5534/3133/85417/567/3845/2793/3106/3725/3442/596/8
/3845/1950/3725/596/90993/7471/3841/1544/595/688/9446/2885/9641/27006
10787/4645/5602/8936/10381/8440/3554/3725

/3442

06/3725/3442/596/2208/9541/1871
201633/22829/3689/3676
58

2/596

5363
